# A systematic interactome of *Saccharomyces cerevisiae* SET1C expands its functional landscape and identifies candidate regulatory connections

**DOI:** 10.7554/eLife.109886

**Published:** 2026-09-16

**Authors:** Pierre Luciano, Kihyun Park, Stephane Audebert, Luc Camoin, Carlos A Niño, Da Kyeong Park, Isabella E Maudlin, Marion Dubarry, Lara Lee, Marlene Oeffinger, Jean D Beggs, Young Hye Kim, Jaehoon Kim, Bernhard Dichtl, Vincent Geli

**Affiliations:** 1 Marseille Cancer Research Center (CRCM), U1068 Inserm, UMR7258 CNRS, Aix Marseille University, Institut Paoli-Calmettes Marseille France; 2 Department of Biological Sciences, Korea Advanced Institute of Science and Technology Daejeon Republic of Korea; 3 Ochang Institute of Biological and Environmental Science, Korea Basic Science Institute Cheongju Republic of Korea; 4 Wellcome Centre for Cell Biology and Institute of Cell Biology, School of Biological Sciences, University of Edinburgh Edinburgh United Kingdom; 5 Institut de recherches cliniques de Montréal, Center for Genetic and Neurological Diseases Montréal Canada; 6 Département de biochimie et médecine moléculaire, Faculté de Médicine, Université de Montréal Montréal Canada; 7 Division of Experimental Medicine, Faculty of Medicine, McGill University Montréal Canada; 8 School of Life and Environmental Sciences, Deakin University Geelong Australia; CABIMER, Universidad de Sevilla Spain; National Cancer Institute United States

**Keywords:** Set1C, interactors, RNA processing, Snf2, methylation, RGG motif, *S. cerevisiae*

## Abstract

Set1 is the catalytic subunit of SET1C or COMPASS, which methylates histone H3K4 and serves as a scaffold for the association of seven tightly bound polypeptides. We have employed yeast two-hybrid screenings to determine the interactome of Set1 and each subunit, providing a unique resource for exploring known and novel roles of the complex. Our screenings identified a multitude of potential interactors involved in chromatin regulation, DNA replication, meiotic breaks, and Ty transposition, processes previously associated with SET1C. Consistent with Set1 being an RNA-binding protein, the screens link SET1C to multiple aspects of RNA biogenesis, including pre-mRNA splicing and polyadenylation. The results reveal that several importins are candidate interactors of Set1, along with RGG motif-containing proteins, providing insights into the mechanisms by which Set1 moves between cytoplasmic and nuclear compartments. We further reveal that reconstituted SET1C interacts with the AT-hook domain of the chromatin remodeler Snf2 and methylates multiple arginines within this domain. In vivo, we report that the ARTSTRGR AT-hook motif is methylated in a Set1-dependent manner, revealing new interplay between lysine and arginine methylation.

## Introduction

Post-translational modifications (PTMs) of histones shape the chromatin landscape and provide essential mechanisms of regulating DNA accessibility, thereby controlling gene expression, genome maintenance, and transmission. Multiprotein complexes of the SET/MLL family methylate histone H3 on lysine 4 (H3K4), which modulates multiple aspects of genome biology (Ruthenburg et al., 2007). Each complex contains additional factors specifying their recruitment to explicit chromatin domains and their specific biological effects (Cenik and Shilatifard, 2021). The budding yeast Set1 complex called COMPASS (for complex of proteins associated with Set1) or SET1C has proved to be an excellent model to study the SET1/MLL family complexes. In *Saccharomyces cerevisiae*, all H3K4 methylation is carried out by SET1C that is composed of Set1, the catalytic subunit, acting as a scaffold for seven other components (Swd1 [RbBP5], Swd2 [WDR82], Swd3 [WDR5], Bre2 [ASHL2], Sdc1 [DPY30], Spp1 [CFP1], and Shg1 [BOD1]) (Nagy et al., 2002; Briggs et al., 2001; Roguev et al., 2001; Miller et al., 2001). Swd1, Swd3, Bre2, and Sdc1 copurify with the SET domain of Set1, yet none of these proteins can interact alone with the SET domain, suggesting that interactions between subunits are required for catalytic domain formation (Kim et al., 2013). The crystal structure of the SET domain of Set1 associated with Swd1, Swd3, Bre2, Sdc1 shows that the catalytic module is organized by Swd1, whose C-terminal tail nucleates Swd3 and a subcomplex formed by Bre2-Sdc1 (Qu et al., 2018; Hsu et al., 2018). For its side, Spp1 associates with the N-SET domain of Set1, while Shg1 binds to the central region of Set1 and Swd2 contacts the N-terminal region of Set1 (Kim et al., 2013; Roguev et al., 2001; Dehé et al., 2006a; Halbach et al., 2009). Swd1 contacts Spp1 and also interacts with the N-terminal region of Set1, suggesting that the C- and N-terminal regions of Set1 may interact with each other (Qu et al., 2018; Acquaviva et al., 2013b; Jeon et al., 2018). Such SET1C organization was confirmed by cryo-electron microscopy and cross-linking experiments performed on the full-length complex (Wang et al., 2018). Interestingly, SET1C has a remarkable mode of assembly that is initiated in the cytoplasm while the nascent Set1 polypeptide emerges from the ribosome. Set1 is initially bound during its translation by Shg1, Spp1, and Swd1; then Swd2, Swd3, Bre2, and Sdc1 associate with the initial pre-complex to form the full SET1C. This explains why Set1 is associated with its own mRNA (Luciano et al., 2017; Halbach et al., 2009).

Swd2, the only essential SET1C subunit, is also part of the APT complex (for Pta1-associated), a subcomplex of the cleavage polyadenylation factor involved in the transcriptional termination of mRNA and snoRNA, suggesting a functional link between SET1C and 3’ end formation/termination (Cheng et al., 2004; Nedea et al., 2003; Dichtl et al., 2004). Set1 has also been linked genetically to the Nrd1-Nab3-Sen1 complex (Kim et al., 2006; Arigo et al., 2006), but intriguingly, it was reported to have two seemingly contradictory effects on ncRNA termination: on the one hand, Set1 was suggested to promote efficient snoRNA termination mediated by Nrd1 (Terzi et al., 2011), and on the other hand, it appeared to interfere with the early termination of a large number of ncRNAs (Castelnuovo et al., 2014; Margaritis et al., 2012). Interestingly, various lysines distributed between Nrd1, Nab3, and Sen1 are methylated, notably lysine 363 in the RNA recognition motif (RRM) of Nab3 whose mono methylation depends on Set1 and Set3 (Lee et al., 2020).

Set1 interacts co-transcriptionally with the RNA polymerase II carboxy-terminal domain (PolII CTD) phosphorylated on Ser5 of the heptad repeats, producing an H3K4 methylation gradient that starts at nucleosome +1 and fades away from the promoter (Soares et al., 2017; Ng et al., 2003). It was recently shown that the N-terminal region of Set1 and Swd2 interact cooperatively with the CTD of Rbp1 (RNA polymerase II large subunit), promoting SET1C recruitment to transcription elongation complexes at the 5′ ends of genes (Bae et al., 2020). Deletion of residues 200–210 of Set1 abolishes interaction with the Rpb1-CTD and Swd2, while deletion of the first 200 amino acids of Set1 strongly reduces trimethylation of H3K4 (H3K4me3) at the 5' end of transcribed genes (Bae et al., 2020). Interestingly, Swd2 is ubiquitinated by Rad6/Bre1, and preventing Swd2 ubiquitination affects H3K4 trimethylation with a concomitant reduction in Spp1 recruitment to chromatin, suggesting a cross-talk between Swd2 and Spp1 (Vitaliano-Prunier et al., 2008). In a similar vein, di- and trimethylation of H3K4 by Set1, which depends on prior ubiquitination of histone H2B by Rad6/Bre1 (Dover et al., 2002; Sun and Allis, 2002), requires a contact between Spp1 and Swd1 (Hsu et al., 2019; Jeon et al., 2018). Other modes of SET1C recruitment to chromatin must exist in the absence of the Set1 N-terminal region, probably via direct interactions between SET1C’s catalytic domain and the nucleosome (Jeon et al., 2018; Dehé and Géli, 2006b; Thornton et al., 2014).

The central region of Set1 contains two RRMs positioned in tandem (Trésaugues et al., 2006) through which Set1 binds directly to RNA. This binding contributes to retain Set1 in the 5' region of genes, thus facilitating their H3K4 trimethylation (Luciano et al., 2017; Battaglia et al., 2017; Sayou et al., 2017). Both the N-SET domain and Spp1 also contribute to Set1 binding to RNA. Set1 associates post-transcriptionally with transcripts produced by specific classes of genes, including snRNA, Ty1, and a number of genes involved in adaptive responses (Luciano et al., 2017). The dRRM itself is flanked by an autoinhibitory region that negatively regulates H3K4me3 (Schlichter and Cairns, 2005). It is possible that in the context of full-length Set1, the alternative mode of SET1C recruitment may be inhibited by the autoinhibitory domain.

In *S. cerevisiae*, H3K4me3 methylation is counteracted by the demethylase Jhd2, a conserved JARID1 family protein (Huang et al., 2010; Liang et al., 2007). The erasure of ancestral histone methylation states results from both active enzymatic demethylation by Jhd2 and passive dilution of parental histones during replication (Radman-Livaja et al., 2010). SET1C is a dimer, and its dimerization depends on the Sdc1 subunit (Choudhury et al., 2019). It has been proposed that the symmetrical methylation of H3K4 on nucleosomes is a consequence of the dimeric nature of SET1C and that Jhd2 preferentially demethylates asymmetrical H3K4me3 (Choudhury et al., 2019). Interestingly, H3K4me3 at environmental stress genes depends on H3-P16 isomerization, a process that controls K4me3 by balancing the actions of Jhd2 and Spp1 (Howe et al., 2014).

The complexity of the *S. cerevisiae* SET1C, the specific arrangement of subunits within the complex, and the specific roles of certain subunits, notably Spp1 and Swd2 (Acquaviva et al., 2013a), raise the question of the contribution of SET1C and of its subunits to multiple processes. Overall, H3K4 methylation, SET1C, and its individual subunits have been involved in multiple processes in *S. cerevisiae,* such as meiotic recombination (He et al., 2019; Sollier et al., 2004; Borde et al., 2009; Acquaviva et al., 2013b; Sommermeyer et al., 2013; Karányi et al., 2018; Adam et al., 2018), stress response, and epigenetic transcriptional memory (D’Urso et al., 2016; Kim and Buratowski, 2009; Weiner et al., 2012; Deshpande et al., 2022; Nadal-Ribelles et al., 2015), DNA repair (Faucher and Wellinger, 2010), telomere and ribosomal DNA (rDNA) silencing (Jezek et al., 2023; Corda et al., 1999; Briggs et al., 2001; Bryk et al., 2002; Santos-Rosa et al., 2004; Nislow et al., 1997), cell wall biogenesis (Nislow et al., 1997), chromosome segregation (Beilharz et al., 2017; Zhang et al., 2000), antisense transcription (Murray et al., 2015; van Dijk et al., 2011; Margaritis et al., 2012; Castelnuovo et al., 2014), transcription termination (Kaczmarek Michaels et al., 2020; Nedea et al., 2003; Dichtl et al., 2004; Cheng et al., 2004; Nedea et al., 2008; Terzi et al., 2011; Soares and Buratowski, 2012; Castelnuovo et al., 2013; Lee and Wang, 2018), Ty silencing (Luciano et al., 2017; Berretta et al., 2008), chronological aging (Gong et al., 2023; Walter et al., 2014; Mei et al., 2019), ergosterol homeostasis (South et al., 2013), lipid homeostasis (Giaever et al., 2019), and DNA replication (Ghaddar et al., 2023; Sollier et al., 2004; Rizzardi et al., 2012; Chong et al., 2020; Delamarre et al., 2020; Santos-Rosa et al., 2021; de La Roche Saint-André and Géli, 2021; Serra-Cardona et al., 2022).

The multiple roles of Set1 and its subunits led us to perform global two-hybrid screening to identify interactors either of Set1-full length or of its N- and C-terminal regions and of each of the individual subunits (Swd2, Shg1, Spp1, Swd1, Swd2, Sdc1, Bre2). The identification of interactors is discussed not only for each bait but also as a whole, revealing new potential functions for the complex. In addition, we demonstrate that the Snf2 AT-hook is methylated by SET1C in vitro. In vivo, deleting *SET1* abrogates arginine methylation within the ARTSTRGR motif of Snf2 AT-hook. These results reveal new interplay between lysine and arginine methylation. This work is an invaluable resource for further exploring known and unsuspected roles of the SET1C complex and its subunits.

## Results

### Identification of Set1 and Set1 subunits interactors

**Figure 1.**
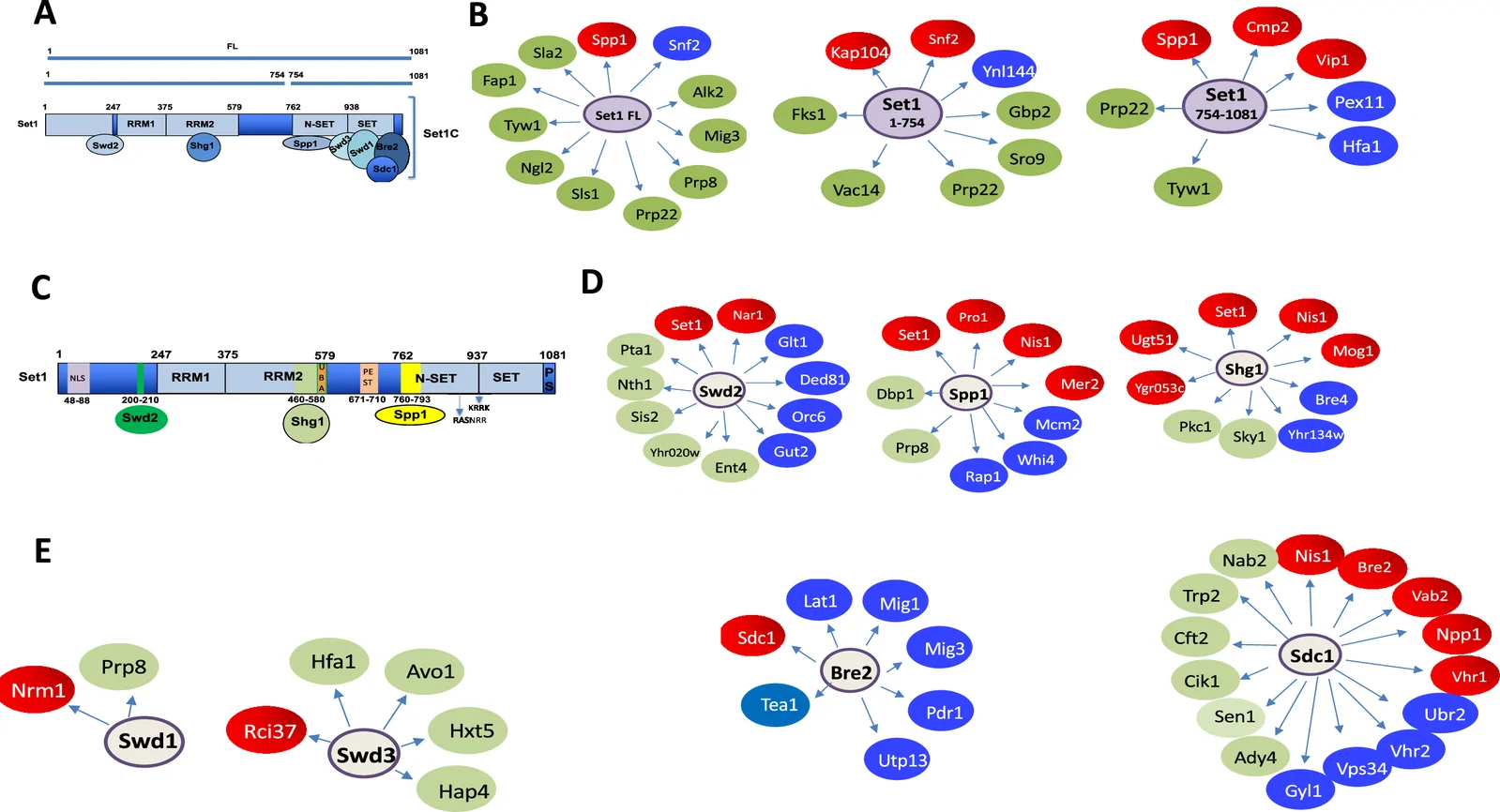
Schematic representation of the Set1 and subunit major interactors identified in the systematic yeast two-hybrid (Y2H) screens. (**A**) Schematic representation of Set1 FL and Set1 fragments 1–754 and 754–1081. (**B**) Set1 FL, 1–754 and 754–1081 major Y2H interactors. (**C**) Interacting regions of Set1 with Swd2, Shg1, and Spp1. (**D**) Swd2, Spp1, Shg1 major Y2H interactors. (**E**) Swd1, Swd3, Bre2, Sdc1 major Y2H interactors. The term ‘interactor’ is used to mean a high-confidence two-hybrid interaction, with the limitations that this entails. The color reflects the predicted biological score (see Materials and methods). Red, highest confidence; blue, high confidence; green, good confidence.

We produced a total of 10 yeast two-hybrid (Y2H) screens (Hybrigenics, see Materials and methods, Supplementary file 1). For the Set1 protein, we performed a total of three screens using as bait the full-length Set1 protein (Set1 FL), the amino-terminal region, including amino acids 1–754, or a C-terminal fragment, including amino acids 754–1081 (Figure 1A). We have chosen to separate Set1 into these two regions because they have been described as having well-defined properties. The Set1 1–754 fragment includes the domain involved in Set1 recruitment to chromatin, the double RRM (Trésaugues et al., 2006), and the central self-inhibitory domain (Schlichter and Cairns, 2005) that all have regulatory roles (Dehé et al., 2006a). The Set1 region 754–1081 contains the N-SET domain, the SET domain, and the post-SET domain: the latter is capable of methylating H3K4 on its own (Thornton et al., 2014; Figure 1—figure supplement 1). For each of the subunits of the complex whose organization is described in Figure 1A, we used the whole protein as bait. Each gene encoding Set1 (and its fragments) or its subunits was fused to the C-terminus of the Gal4-BD except for Swd2 that was fused to the N-terminus of Gal4-BD. These screens have proven their power and effectiveness. In particular, they previously identified Mer2 as an interactor of Spp1 (Acquaviva et al., 2013b), and the CTD of Rpb1 (Rpo21 in Supplementary file 2) as an interactor of the N-terminal region of Set1 (Bae et al., 2020). The results of the 10 Y2H screens are presented in Supplementary file 2. This table also highlights interactors that are shared among multiple subunits, as well as those common to different Set1 fragments. Y2H interactions indicate potential protein-protein associations but do not constitute definitive proof of interaction; accordingly, the list of candidates may include false positives. Each interactor is characterized with a confidence score based on several criteria, including the frequency with which a prey protein is found for a particular screen and the presence of overlapping fragments, which allow the delineation of the interaction domain involved (see Materials and methods). Very high-confidence interactors (indicated by a color code) are likely to interact directly with their bait. For Set1 and its N- and C-terminal fragments, the high-confidence Y2H interactors are shown in Figure 1B. The predicted protein-protein interactions within the Set1 interactors reveal functional information that is detailed in the following paragraphs. The high-confidence interactors of the seven SET1C subunits are shown in Figure 1C–E. We found that Spp1, Shg1, and Swd2 interact alone with Set1 (Figure 1C). The minimum Set1 region for which an interaction is found for each of these three subunits is shown in Figure 1C. The high-confidence interactors of the seven SET1C subunits are shown in Figure 1C–E. We found that Spp1, Shg1, and Swd2 display Y2H interactions with Set1 (Figure 1C). The high-confidence interactors of Spp1, Shg1, and Swd2 are indicated in Figure 1D (see also Supplementary file 2). In contrast, we did not find any interaction between Swd1, Swd3, Bre2, and Sdc1 (SET-c components) and Set1 in the various Y2H screens, suggesting that these subunits must cooperate to bind to Set1. Only Bre2 was found to interact with Sdc1 in both Sdc1 and Bre2 Y2H screens (Figure 1E). These results are consistent with the structure of the extended SET1C catalytic module and full-length SET1C (Wang et al., 2018; Hsu et al., 2018; Qu et al., 2018).

**Figure 1—figure supplement 1.**
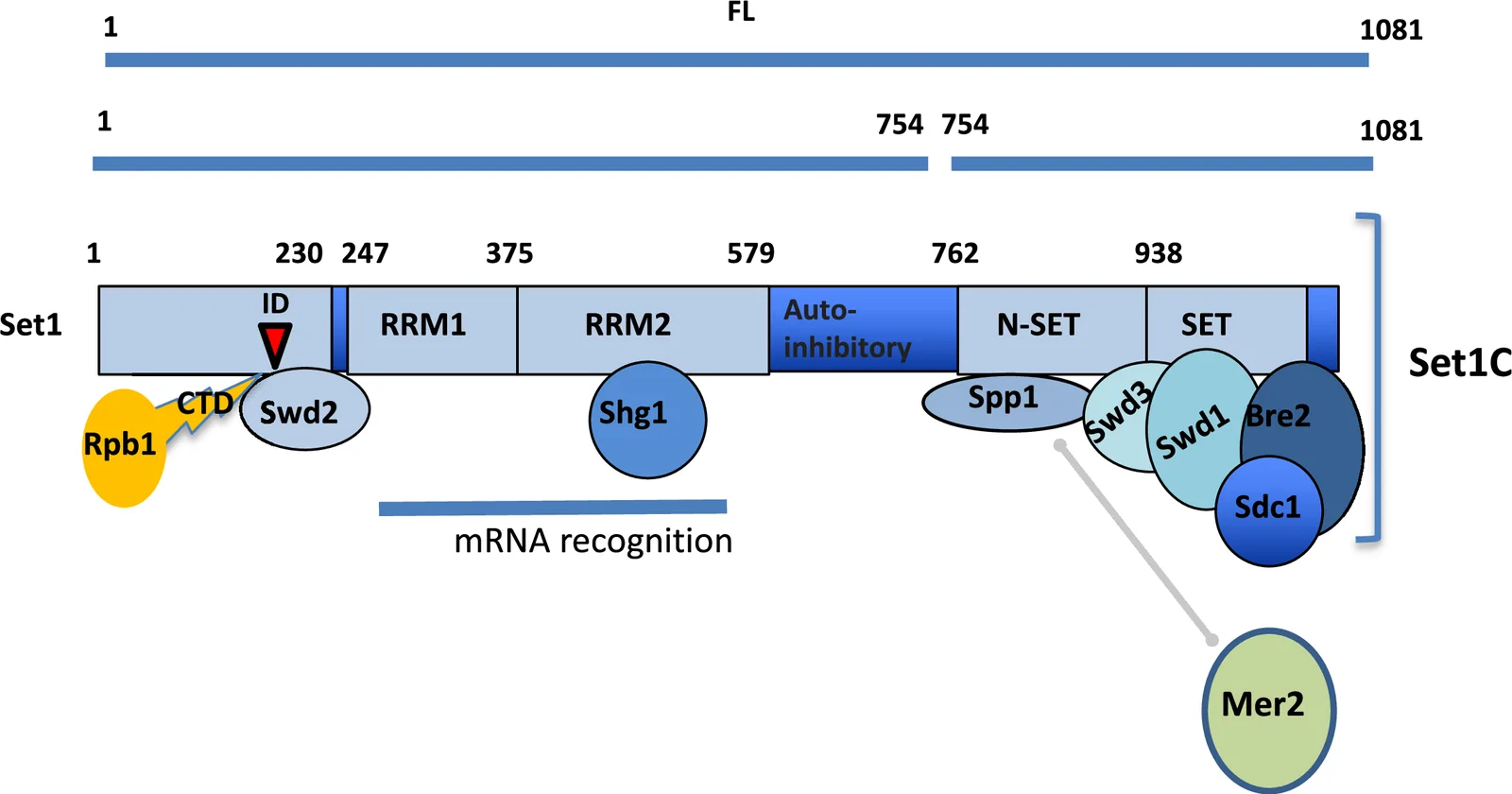
Schematic representation of SET1C/COMPASS. These yeast two-hybrid (Y2H) screens revealed the interaction of Mer2 with Spp1 (Acquaviva et al., 2013a) and of Rbp1-CTD with the N-terminal region of Set1 (Bae et al., 2020).

### Set1 1–754 interacts with the importin Kap104

**Figure 2.**
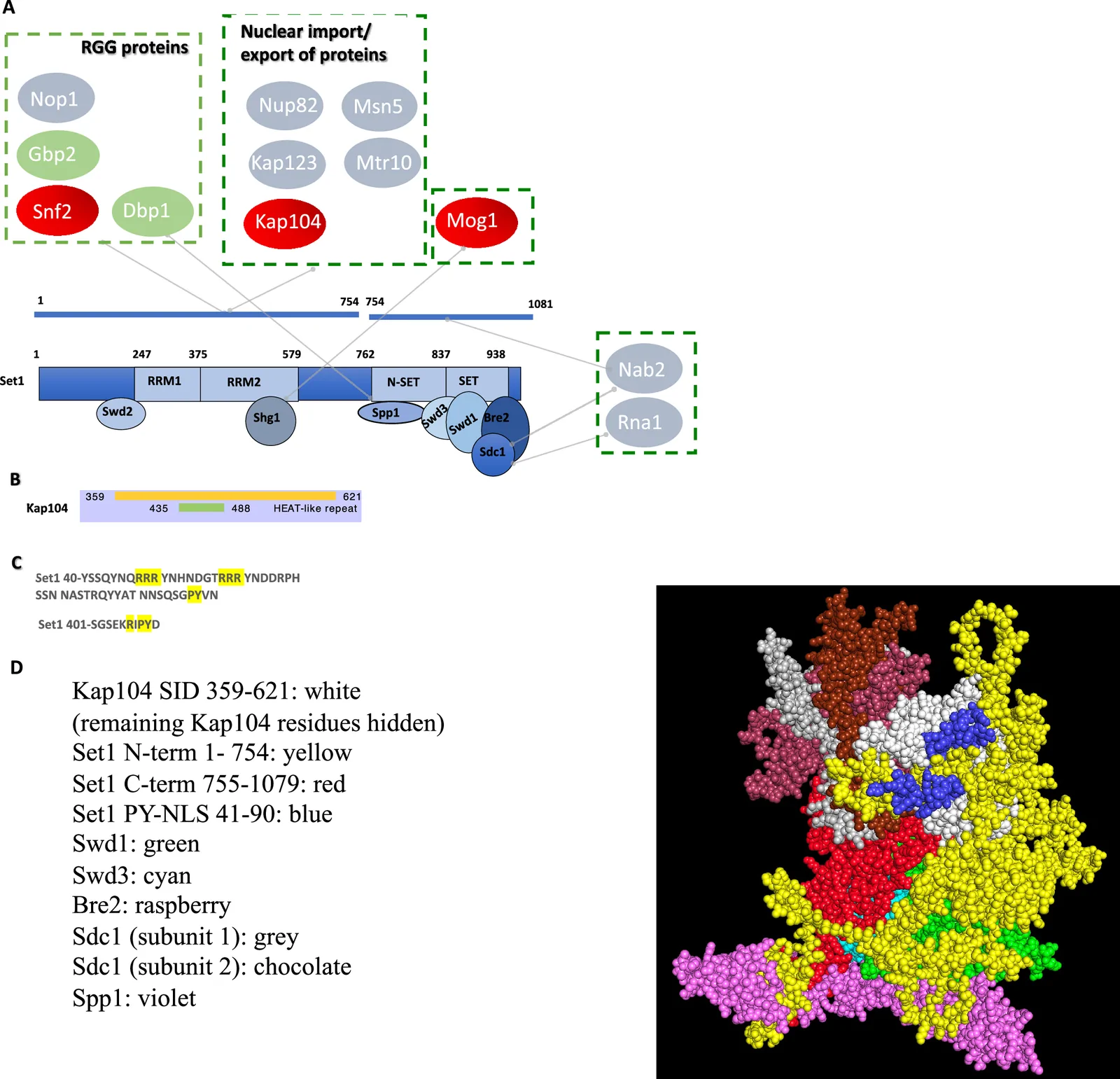
Set1 1–754 interacts with RGG proteins and the importin Kap104. (**A**) RGG proteins and import/export proteins interacting with Set1 1–754, Set1 754–1081, and Spp1, Shg1, and Sdc1. (**B**) Set1 interacting domain (SID) (blue) within Kap104. Heat-like repeats 9 and 10 are represented in purple. (**C**) PY-NLS in the N-terminal region of Set1. (**D**) AlphaFold modeling of a seven subunit Set1C (Set1-Bre2-Sdc1(x2)-Swd1-Swd3-Spp1) and Kap104. A representative model is shown using the following color code: Kap104 SID 359–621 (white, the remaining Kap104 residues are hidden). Set1 N-term 1–754 (yellow), Set1 C-term 755–1079 (red); Set1 PY-NLS 41–90 (blue); Swd1 (green); Swd3 (cyan); Bre2 (raspberry); Sdc1 (subunit 1, gray); Sdc1 (subunit 2, chocolate); Spp1 (violet).

We found that Set1 1–754 interacted with very high confidence with the importin Kap104, suggesting a direct interaction (Figure 2A). Kap104 has been reported to recognize specific cargos containing a specific class of NLS termed PY-NLS (Soniat et al., 2013; Xu et al., 2010). This PY-NLS includes an N-terminal or central hydrophobic or basic motif, which often contains hydrophobic (R/H/K) residues and which can include RGG repeats (arginine-glycine-glycine motifs) and a C-terminal proline-tyrosine (PY) dipeptide near the C-terminus. Such cargos include the mRNA export factor Nab2, the subunit of the THO/TREX complex Hrp1, and the transcription factor Tfg2 (Lee and Aitchison, 1999; Süel et al., 2008). Interestingly, Nab2 is a confident interactor of Sdc1 (Figure 2A). The Set1 interacting domain (SID) of Kap104 extends from residues 359–621 and includes the HEAT-like repeat (Yoshimura and Hirano, 2016; Figure 2B). Consistent with this result, we identified two PY-NLS in the N-terminal region of Set1 that are likely recognized by Kap104 (Figure 2C). To strengthen this observation, we performed AlphaFold modeling of a seven subunit Set1C (Set1-Bre2-Sdc1(x2)-Swd1-Swd3-Spp1) and Kap104 (Figure 2D). Obtained models showed prediction parameters which are indicative of an overall predicted fold for the complex that was at the threshold for a true structure (pTM = 0.53) and below the confidence threshold for predicting the relative positions of the subunits within the complex (ipTM = 0.5). Analysis of a model in Pymol revealed Kap104 SID binding to Set1–754 as predicted from the Y2H (in the structure we only show the Kap104 SID, while the remaining Kap104 sequences are hidden such that the Set1 PY-NLS is visible). In agreement with Y2H data, the model recapitulates Kap104 SID binding to the PY-NLS sequence of Set1 at position 40–90; the second PY-NLS of Set1 is not visible in the shown structure and is not interacting with Kap104 in this model. Moreover, the Spp1 and Sdc1-dimer bind on opposing ends of the complex as expected (Qu et al., 2018). Taken together, the AlphaFold model recapitulates key predictions for the Set1-Kap104 Y2H interaction.

Shg1, which binds RRM2, interacted with very high confidence with Mog1 (Oliete-Calvo et al., 2018). Mog1 has been involved in the modulation of the nucleotide state of Ran-GTP in the nucleus and of Ran-GDP in the cytoplasm, thereby conferring directionality to the nuclear import pathway (Baker et al., 2001). Mog1 has been reported to interact directly with the Ran homologue Gsp1 (Oki and Nishimoto, 1998; Figure 2A). We have also identified Kap123, Msn5 (Kap142), Nup82, and Mtr10 as interactors of Set1 1–754 that are also involved in protein import/export (Figure 2A). Kap123 is a major karyopherin that recognizes NLS of cytoplasmic H3 and H4 (An et al., 2017), while Msn5/Kap142 was shown to mediate the import into the nucleus of the subunits of RPA (Yoshida and Blobel, 2001). Nup82, for its part, belongs to a module at the cytoplasmic face of the NPC and interacts with karyopherins (Beck and Hurt, 2017; Figure 2A). Finally, Mtr10 has been implicated in the nuclear import of the mRNA-binding protein Npl3 (Senger et al., 1998; Pemberton et al., 1997). Collectively, these multiple interactions reveal insights for understanding the nuclear import of Set1, which was reported to bind co-translationally with Shg1, Swd1, and Spp1 (Halbach et al., 2009).

### Set1 1–754 interacts with Snf2 and RGG motif proteins

Set1 1–754 and Set1 FL interacted in the 2H screens with very high confidence with Snf2, the catalytic subunit of the SWI/SNF chromatin remodeling complex (Côté et al., 1994; Figure 1B, Supplementary file 2). Interestingly, two additional Snf2 complex components, Snf5 and Swi1, have been identified in the Set1FL screen (Supplementary file 2). Together, these observations strongly support the idea that the suggested interaction of Set1 and SWI/SNF is of biological significance. The various Y2H screens revealed Set1 and its subunits interacted with a number of proteins involved in chromatin structure regulation, although the relevance of these interactions remains to be demonstrated (Supplementary file 2, Appendix 1—figure 1). Interestingly, the SID region of Snf2 is juxtaposed to an RGG motif composed of several RGG/RG repeats that is often found in RNA-binding proteins (Thandapani et al., 2013) and may be the substrate for arginine methyltransferases (McBride et al., 2005). RGG repeats are multifunctional motifs that mediate RNA and DNA binding and nuclear import. They have been involved in RNA metabolism and chromatin dynamics, possibly via arginine methylation, which modulates RNA affinity and nuclear localization (108). Along the same line, Set1 1–754 also interacted with the mRNA export factor Gbp2 (Poornima et al., 2021) and the nucleolar protein Nop1, both of which contain an RGG motif (Appendix 1—figure 2). Other RGG proteins, such as polyadenylated RNA-binding protein Nab2 and the RNA-helicase Dbp1, interacted with Set1 754–1081 (and Sdc1) and Spp1, respectively (Appendix 1—figure 2). For each of these proteins, the putative SID includes the RGG motif. Of note, both Nop1 and Nab2 are methylated by the arginine methyltransferase Hmt1 (Smith et al., 2020; Green et al., 2002).

### SET1C is involved in multiple aspects of RNA biogenesis

**Figure 3.**
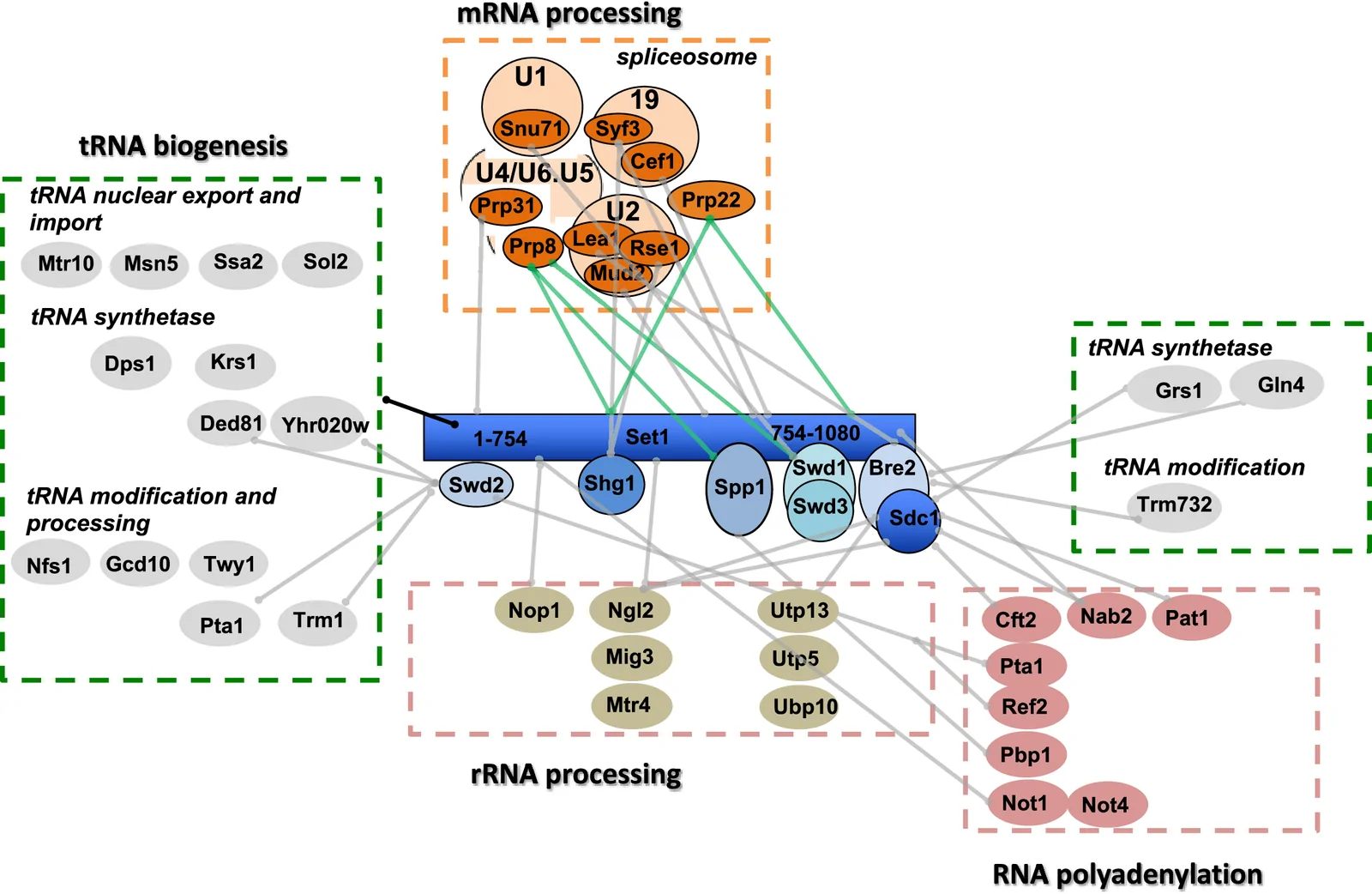
The SET1C yeast two-hybrid (Y2H) interactome identifies proteins involved in RNA biogenesis. All Y2H interactors are described in Supplementary file 2. The processes linked to RNA metabolism in which the different interactors are involved are shown in the figure. The green lines linking Prp22 to Set1FL/Set1 754–1080 and Prp8 to Set1 FL and Spp1 indicate interactions with a high degree of confidence.

Set1 was previously shown to bind RNA nascent transcripts through its dRRM, contributing to position Set1 and H3K4me3 predominantly to the 5′ regions of genes. Of note, Set1 showed a higher occupancy within introns, at transcripts from rDNA and tRNAs (Luciano et al., 2017; Battaglia et al., 2017; Sayou et al., 2017). Moreover, Set1 also binds post-transcriptionally to Ty1 retrotransposon transcripts and mRNA encoding specific transcription factor genes (Luciano et al., 2017). The SET1C Y2H interactome revealed that many proteins involved in RNA biogenesis were candidate interactors, interacting directly or indirectly with Set1 or its subunits (Figure 3). It is not feasible to validate all of these interactions within the limits of this manuscript, and their validity should therefore be interpreted with caution. Nonetheless, these findings provide a useful basis for future research. We find a strong connection between SET1C and pre-mRNA processing, as highlighted by the identification of multiple spliceosome subunits (Figure 3). Some spliceosome subunits were found in several screens (Figure 1B and D; Supplementary file 2). For instance, the Prp8 subunit of the U5 snRNP that functions in critical molecular rearrangements during the splicing process (Grainger and Beggs, 2005) interacted with Set1 FL, Spp1, and Swd1. Similarly, Prp22 interacts with Set1FL and Set1 754–1080. Prp22 is a DEAH-box helicase that associates with newly spliced mRNA and promotes its release from the spliceosome (Will and Lührmann, 2011).

**Figure 4.**
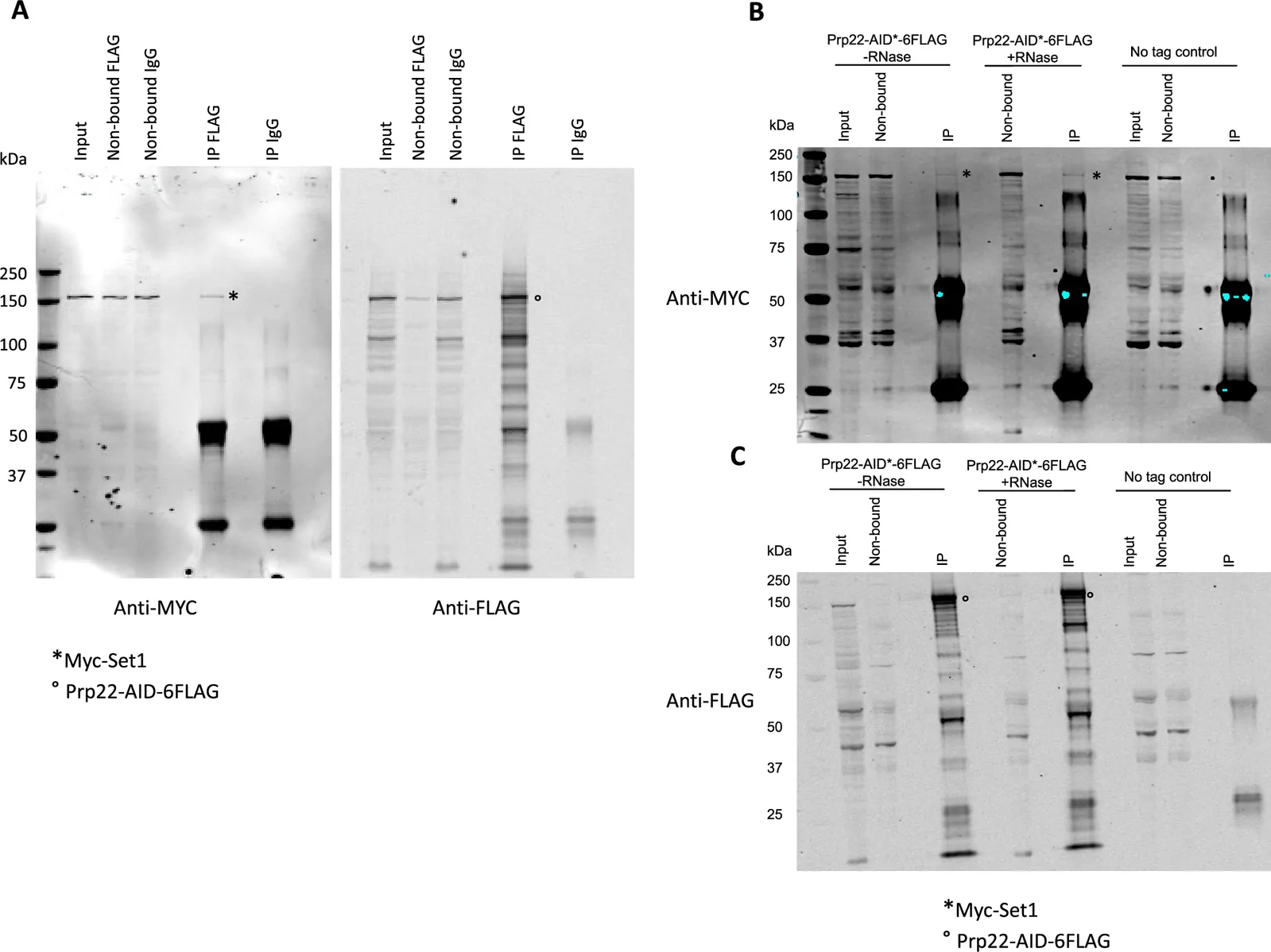
Set1 is co-precipitated with Prp22 in vivo. (**A**) Co-immunoprecipitation experiments were performed in W303 expressing chromosomally encoded Myc-Set1 (Dehé et al., 2006a) and Prp22AID-FLAG (Mendoza-Ochoa et al., 2019). Prp22-AID-6FLAG was pulled down using a FLAG antibody. IgG was used as a negative control. Input, non-bound, and immunoprecipitation (IP) samples were loaded. The western blot was probed either with an antibody against the MYC epitope tag (left) or against the FLAG epitope tag (right). Myc-Set1 and Prp22AID-FLAG are indicated by * and °, respectively. (**B, C**) The same experiment was carried out in the absence or presence of RNase. A strain with 9MYC-Set1 and no flag-tagged Prp22 was used as a negative control. The western blots were probed with anti-MYC and anti-FLAG, respectively. Figure 4—source data 1.Source data for the co-immunoprecipitation experiments shown in Figure 4.

We have further strengthened the link between Set1 and Prp22 by showing that Set1 is co-immunoprecipitated with Prp22 in vivo in an RNA-independent manner (Figure 4). The preferential binding of Set1 to genes with introns (Luciano et al., 2017) and its interaction with splicing factors, in particular Prp22, suggest that Set1 may be involved in late splicing events. Alternatively, Prp22 and/or other splicing factors could be involved in H3K4 methylation.

We also found a number of factors involved in rRNA processing that could be related to the binding of Set1 to ncRNA transcripts derived from the rDNA intergenic spacer regions (Sayou et al., 2017; Figure 3). Next, and consistent with the observation that SET1C regulates the choice of the polyadenylation site and the recruitment of the cleavage/polyadenylation complex (Kaczmarek Michaels et al., 2020), the Y2H screens revealed multiple potential interactions between SET1C and factors involved in RNA polyadenylation (Figure 3). Some of these interactions are known, such as the interactions between Swd2 with Pta1 and Ref2, all three proteins belonging to the APT complex (Nedea et al., 2008). Interestingly, we found that Not1 and Not4 that belong to the ubiquitin-protein ligase CCR4-NOT (Liu et al., 2001) were potential interactors of Set1 1–754 (Figure 3). This complex was shown to be involved in the regulation of H3K4me3 via a ubiquitin-dependent pathway (Mulder et al., 2007; Laribee et al., 2007) that was subsequently linked to Jhd2 degradation (Huang et al., 2010; Mersman et al., 2009). These results raise the question of which of the Y2H interactions described in this study are linked to the regulation of H3K4 methylation states.

Finally, and somewhat surprisingly, we found that many Y2H interactors of Set1 1–754 were involved in tRNA nuclear transport, modification, and synthesis (Figure 3). Swd2, which interacts with the Set1 N-terminus, exhibits a 2H interaction with a high degree of confidence with the cytoplasmic asparaginyl-tRNA synthetase Ded81 and the prolyl-tRNA synthetase Yhr020, while Sdc1 interacted with the glycyl-tRNA synthase Grs1 and Bre2 with the glutamine tRNA synthetase Gln4. Of note, Trm1 (Liu et al., 1998) and Trm732 (Guy et al., 2012) are both directly or indirectly involved in tRNA methylation and form part of what has been defined as the cell’s global methyltransferome (Giaever et al., 2019).

### SET1C Y2H interactors are involved in several aspects of DNA transactions

As mentioned in the Introduction, it has been shown that SET1C, and in particular Spp1, regulates the selection of meiotic breaks, the progression of the replication fork, DNA repair, chromosome segregation, and transposition of Ty elements (Deshpande and Bryk, 2023). We have classified all Set1 and subunit interactors according to these SET1C roles. We found a number of putative interactors involved in meiosis. In particular, Spp1 not only interacts with Mer2, but may also associate with the meiosis-specific DNA helicase Mer3 (Nakagawa and Kolodner, 2002), which itself may interact also with Swd1 (Appendix 1—figure 3). We further found Set1 candidate interactions with the kinetochore proteins Spc25 and Cbf2, suggesting that Set1 could be transiently localized at the spindle pole body. Regarding the biology of retrotransposons, we previously reported that Set1 binds post-transcriptionally to Ty1 mRNA and represses Ty1 mobility (Luciano et al., 2017). Interestingly, the two-hybrid screens reveal that Set1 1–754 interacted with Gag capsid-like proteins of Ty1 (Appendix 1—figure 3), raising the possibility that Set1 binding to Ty1 mRNA is linked to the interaction of Set1 1–754 with Gag. Concerning the role of Set1 and subunits in DNA replication and repair, we report multiple potential interactions (Appendix 1—figure 3). In particular, Swd2, Swd1, and Spp1 exhibited a high-confidence 2H interaction with Orc6, Nrm1, and Mcm2, respectively (Supplementary file 2 and Appendix 1—figure 3). Orc2 was previously described to interact physically with Spp1 (Kan et al., 2008). We recently reported that Spp1 is recruited at replication forks stalled at the Tus/Ter barrier independently of its interaction with Set1 (Ghaddar et al., 2023). Interestingly, in the Y2H screen, Spp1 interacted with the extreme C-terminal region of Mcm2 (791–867) (Appendix 1—figure 4A) that corresponds to a non-conserved accessible alpha-helix within the MCM complex (Li et al., 2015b). We fused Spp1 and Mcm2 to GST and performed GST pull-down experiments. We confirmed in vitro in both directions a weak interaction between Spp1 and Mcm2 (Appendix 1—figure 4B). Whether Spp1 is recruited by Mcm2 at stalled replication forks remains to be determined.

Screening with *SWD1* yielded a high number of clones, 14 of which contained fragments of the *NRM1/YNR009w* gene, which encodes a basic protein of 249 amino acids (Supplementary file 2). Nrm1 inactivates MBF, a major regulator of the G1/S transcription (de Bruin et al., 2006). During replication stress, Nrm1 phosphorylation by the checkpoint kinase prevents its binding to MBF target promoters, leading to the activation of G1/S transcription (Travesa et al., 2012). An exciting idea is that Swd1 recruits Nrm1 to stalled forks by promoting its phosphorylation by Rad53. Swd1 would play a role in linking replication stress and transcriptional regulation via Nrm1. Of note, Nrm1 was identified as a gene required for the cell-cycle pattern of H3K79me2 during early S phase (Schulze et al., 2009). Interestingly, Nrm1 exhibits an H3K4-like domain and raised our attention to other yeast proteins that carry such sequences. We used the scansite search algorithm (http://scansite.mit.edu) to systematically identify sequence motifs that are related to the SET1C modification site in histone H3. Search parameters included four to six identical residues of the ARTKQT sequence that is found at the H3K4 modification site (Supplementary file 3). In addition, we did allow for biochemically equivalent amino acid changes. Appendix 1—table 1 shows eight candidate proteins based on their function and the sequence context of the H3K4-like domain. Some of these gene products are involved in cellular pathways that functionally overlap with SET1C, e.g., transcription (Not5), rDNA silencing (Irs4), and cell-cycle control (Dbf2 and Dbf20), increasing the possibility of occurring physical and functional interactions.

Finally, Y2H screening indicated that Set1 and its subunits exhibit 2H-interactions with a number of proteins involved in protein SUMOylation (Appendix 1—figure 3). The proteins were either involved in SUMO conjugation or SUMO-dependent degradation. Remarkably, the C-terminus of Nis1 (360–407) that contains a potential SUMO-binding site (Hannich et al., 2005) was identified as a high-confidence interactor of Spp1, Shg1, and Sdc1 (Figure 1). Nis1 is localized at the bud neck (Iwase and Toh-e, 2001) at the vicinity of the septin collar containing several highly SUMOylated proteins (Shs1, Cdc11) (Wykoff and O’Shea, 2005) and has been implicated in preventing bud recovery at the site of division (Meitinger et al., 2014). We thus sought to confirm biochemically the interaction of Nis1 with the three SET1C subunits. We fused the GST to Spp1, Sdc1, and Sgh1 to perform pull-down experiments with in vitro translated Nis1. We confirmed the interaction of Nis1 with Spp1 and Sdc1, but not with Sgh1 (Appendix 1—figure 5). However, mass spectrometry analyses on TAP-Nis1 did not reveal the presence of SET1C subunits (Supplementary file 4), suggesting that interaction between Nis1 and Spp1/Sdc1 might be transient. The relevance of the Nis1 and other putative interactors remains unclear, especially as many of those proteins are not known to be located in the nucleus. Interestingly, Nis1 has been reported to shuttle from the bud neck to the nucleus when overexpressed (Perez and Thorner, 2019). In Appendix 1—figure 6, we have identified a number of interactors, including Sdc1 and Spp1, showing changes in localization following hypoxia (Henke et al., 2011). Of note, one of the two human SET1C homologs, SET1B, has a cytoplasmic location with functions unrelated to H3K4 methylation (Wang et al., 2017). Combined, these observations raise the question of the minor or transient localization of Set1 and its subunits outside the nucleus, or conversely of the transient localization of interactors within the nucleus.

### Set1 is SUMOylated

**Figure 5.**
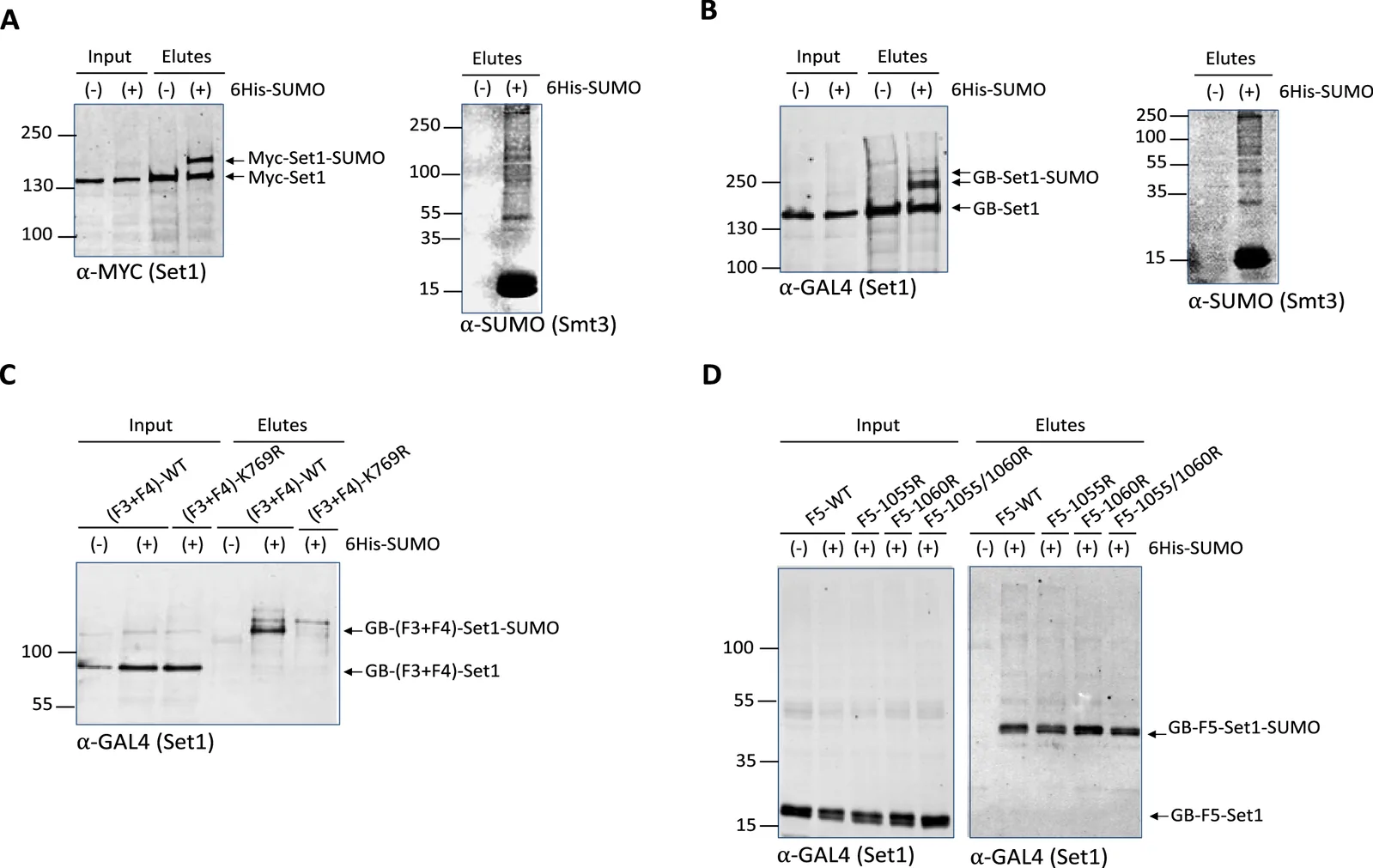
Set1 is SUMOylated. 6His-SUMO-conjugated proteins were purified from cells transformed (+) or not transformed (−) with a plasmid encoding 6His-SUMO under control of the *CUP1* promoter. Cell lysates (input) and Ni-purified material (elutes) were analyzed by western blotting with an anti-MYC antibody (**A**) or an anti-GAL4 antibody (**B–D**). Analysis of 6His-SUMO-conjugated forms of (**A**) genomically MYC-tagged Set1 or (**B**) GB-Set1 transformed cells was performed (left panels); in both cases, SUMO expression and efficiency of purification were controlled using an anti-SUMO antibody (right panels). (**C**) SUMOylation analysis of Set1 fragment F3+F4 (aa. 351–956) wild-type (WT) and the K769R mutant. (**D**) SUMOylation analysis of Set1 fragment F5 (aa. 956–1080) WT, single mutants K1055R and K1060R, and the double mutant K1055R/K1060R mutant. Unmodified MYC-Set1 and GAL4-Set1 in both the (–) and (+) His-SUMO eluates are most likely due to the stickiness of unmodified Set1 to the beads. Figure 5—source data 1.Source data for the western blots shown in Figure 5.

Global analyses of SUMOylated proteins in fission yeast revealed that Set1 and Spf1 (Spp1) were SUMOylated (Shin et al., 2005; Nie et al., 2015). As Set1 had two-hybrid interactions with Slx5 and Wss1, both of which act on SUMOylated proteins as they undergo protein degradation (Mullen et al., 2010), we tested whether Set1 can be SUMOylated. Cells expressing Myc-Set1 (Dehé et al., 2006a) or transformed with pPB66-SET1 expressing GBD-Set1-FL (GAL4 binding domain) were transformed with a plasmid encoding His_6_-SUMO or a control plasmid. Proteins were purified on Ni-NTA agarose beads and detected by western blot either using anti-MYC or anti-GAL4 antibodies. The results revealed that Set1 can be mono-SUMOylated (Figure 5A) or di-SUMOylated (Figure 5B). This difference may be due to the fact that GBD-SET1-FL is under the control of the ADH promoter in plasmid pGB66 and thus overexpressed.

**Figure 5—figure supplement 1.**
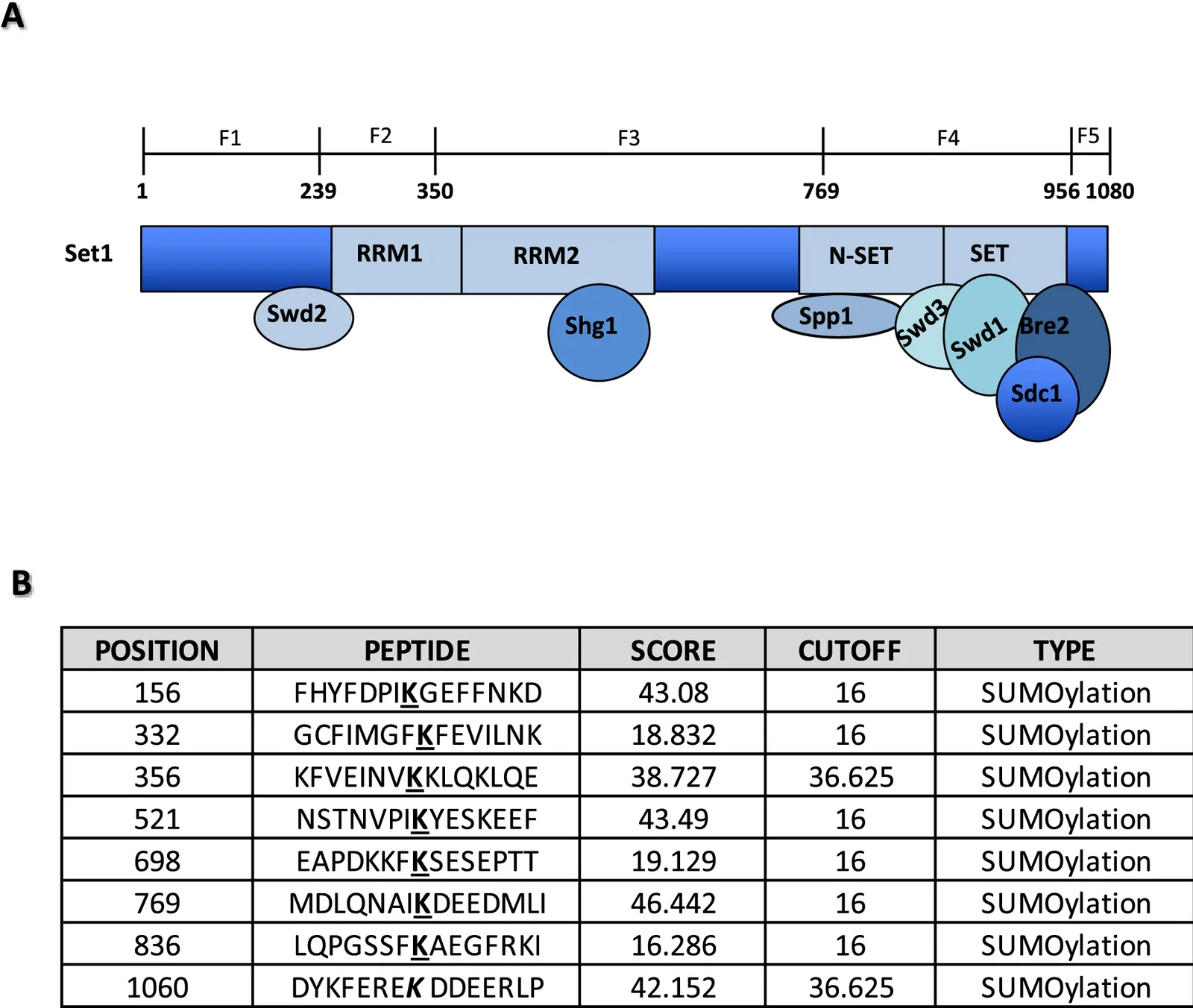
Set1 SUMOylation analysis. (**A**) Schematic representation of the F1-F5 Set1 fragments. (**B**) List of predicted SUMOylated sites by GPS-SUMO 1.0 (Zhao et al., 2014). The predicted SUMOylated lysine is bold underlined.

We sought to refine the SUMOylated Set1 region. We transformed the plasmid encoding His6-SUMO and the control plasmid into cells expressing Set1 fragments F1, F2, F3, F4, F3+F4, and F5 (Figure 5—figure supplement 1A). We expressed F3+F4 instead of the isolated F3 and F4 fragments in order to preserve the K769-centered motif predicted to be highly SUMOylated (Figure 5—figure supplement 1B). F1, F2, F3, and F4 were not SUMOylated (not shown) while the F3+F4 fragment was clearly SUMOylated (Figure 5C). In order to identify whether K769 is important for F3+F4 SUMOylation, we mutated K769 to R. We show that introduction of K769R into F3+F4 abolishes its SUMOylation (Figure 5C), indicating that K769 is likely to be the SUMOylated lysine within the F3+F4 fragment. We then found that F5 was also SUMOylated (Figure 5D). In this case, the substitution K1055R, K1060R, or the double substitution does not affect the SUMOylation of F5, indicating that this motif, also predicted to be SUMOylated with a high score, is not the SUMOylated motif (Figure 5D). Taken together, these experiments indicate that Set1 can be SUMOylated in the N-SET and SET domains, in the interaction region of Spp1 and Swd1-Swd2-Bre2-Sdc1, respectively. This raises the possibility that SUMOylation regulates the interaction of Set1 with its subunits, in particular with Spp1, which interacts dynamically with SET1C (Ghaddar et al., 2023; D’Urso et al., 2016; Karányi et al., 2018; Serra-Cardona et al., 2022). Interestingly, in mammals, the SUMO peptidase SENP3 (the ortholog of Ulp1 in budding yeast) interacts with MLL1 and MLL2, catalyzing the deSUMOylation of RbBP5 (Swd1). This process regulates the association of specific subunits of MLL1/MLL2, such as menin and Ash2L (Bre2), with the DLX3 gene, which plays a role in osteogenic differentiation (Nayak et al., 2014).

### SET1C Y2H interactors regulate metabolism and stress responses

Set1 was reported to regulate ergosterol levels (South et al., 2013). We identified Erg9, Ugt51, Vhr1, Vhr2, and Ste20, all involved in ergosterol metabolism (Daicho et al., 2020; Lees et al., 1995; Warnecke and Heinz, 1994). Erg9 and Ste20 were candidate interactors with Set1 while Ugt51 and Vhr1/Vhr2 were high-confidence 2H interactors of Shg1 and Sdc1, respectively (Appendix 1—figure 7). Along the same line, the various Y2H screens revealed numerous genes involved in phosphatidyl inositol metabolism as potential interactors of Set1 and its subunits. In particular, Vip1 is a high-confidence 2H interactor of Set1 754–1081 (Figure 1B). Vip1 is a bifunctional inositol pyrophosphate kinase and phosphatase that regulates IP7 levels in the inositol pyrophosphate (PP-IP) synthesis pathway (Lee et al., 2007; Mulugu et al., 2007). Interestingly, Vip1 was reported to regulate the environmental stress response (ESR) through IP7 that activates the HDAC Rdp3 (Worley et al., 2013), suggesting potential new avenues to explain ESR regulation by Set1 (Weiner et al., 2012). Along the same line, Swd1 and Swd3 were identified in a screen aimed to identify genes that negatively regulate the PHO pathway in a Vip1-dependent manner (Choi et al., 2017). How the putative interaction between Set1 754–1081, which also interacts with the Pho23 subunit of the Rpd3L complex, and Vip1 fits into these processes remains to be discovered. Of note, Vip1 has been shown in *Arabidopsis thaliana* to change localization upon hypo-osmotic stress from the cytosol to the nucleus (Takeo and Ito, 2017). On their side, Y2H interactors identified as stress-responsive genes interact either with Set1 1–754 or Swd2 (Appendix 1—figure 7). Swd2 interacted with high confidence with Nar1, an essential Fe/S protein required for the assembly of cytosolic Fe/S proteins (Balk et al., 2004) and the calcineurin phosphatase Cmp2 activated in response to ER stress (Mizuno et al., 2018). In contrast to the stress genes mentioned above, many interactors involved in glucose metabolism (repression) interact only with the Set1 1–1081 or with members of the nSET module (Appendix 1—figure 7).

### Reconstituted Set1C methylates the Snf2 RG motif within the AT-hook in vitro

**Figure 6.**
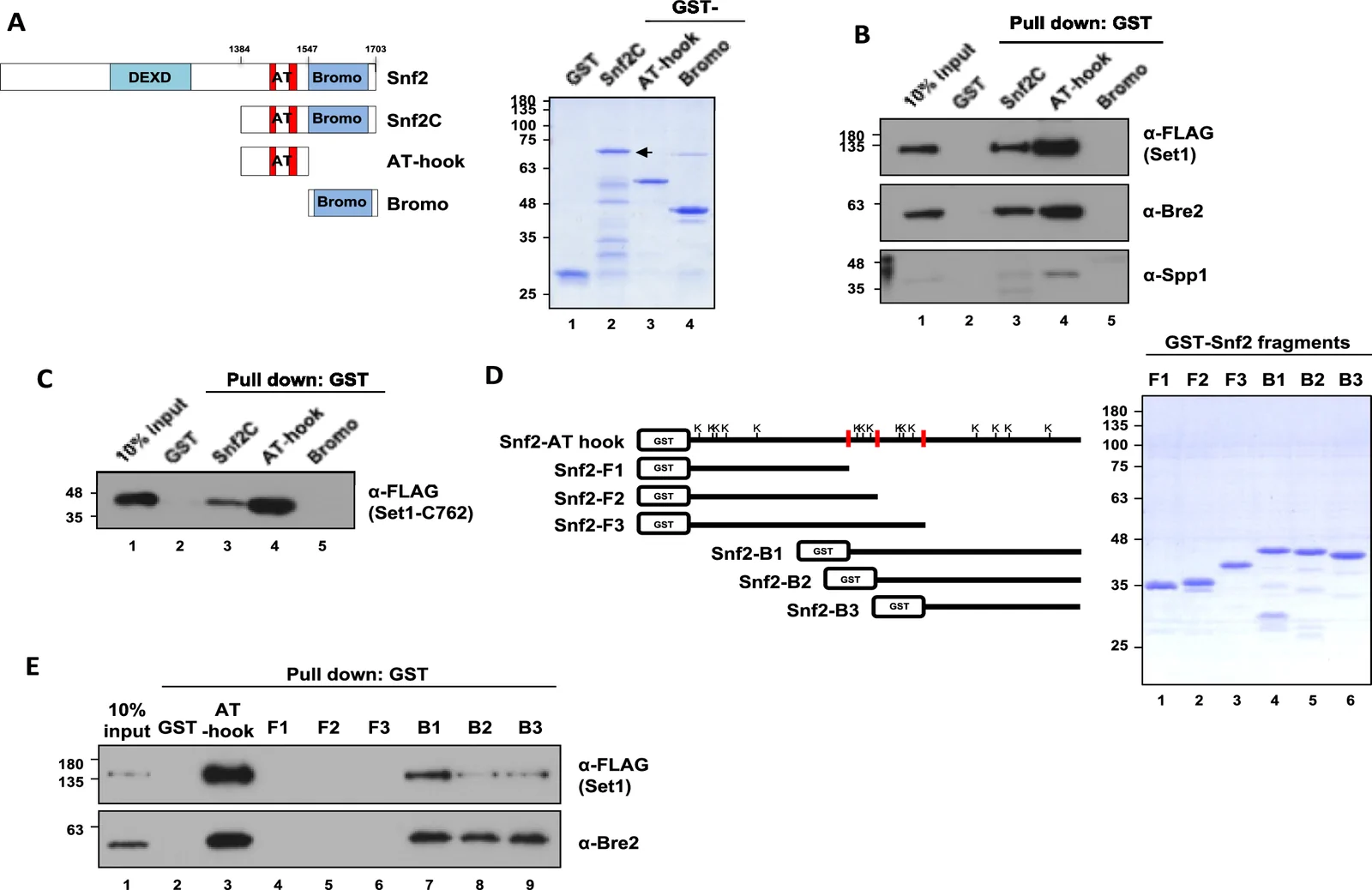
SET1C interacts in vitro with Snf2C-AT-hook. (**A**) A schematic diagram depicting the domains of Snf2 and the Snf2 fragments used in this study, along with the SDS-PAGE/Coomassie staining of the purified GST-tagged Snf2 fragments. (**B, C**) GST pull-down assay using purified GST-tagged Snf2 fragments. The purified SET1C (**B**) or SET1C-C762 complex (**C**) was mixed with GST-tagged Snf2 fragments, followed by GST pull-down, and the bound proteins were analyzed by immunoblotting. (**D**) A schematic diagram illustrating Snf2 fragments with a more detailed breakdown of the AT-hook domain, along with the SDS-PAGE/Coomassie Blue staining of the purified Snf2 fragments. The lysines present in the AT-hook are represented by the letter K. (**E**) GST pull-down assay using purified GST-tagged Snf2 fragments and SET1C. Figure 6—source data 1.Source data for the Coomassie blue–stained gels, western blot, and autoradiographs presented in Figure 6. Figure 6—source data 2.Uncropped and labelled gel image corresponding to Figure 6.

Our screens indicate that Snf2 (residues 1430–1499) was potentially interacting with Set1 1–754 (Supplementary file 2). To validate this interaction, we obtained recombinant protein where GST was fused to the C-terminal domain of Snf2 (Snf2C), the AT-hook (Snf2C-AT-hook), and the Bromo domain (Snf2C-Bromo) (Kim et al., 2010; Figure 6A). We performed pull-down assays to test the interaction of the GST-fusion proteins with reconstituted SET1C or with the SET1C762 complex in which Set1 has an N-terminal truncation of the first 761 residues, respectively (see Materials and methods) (Figure 6—figure supplement 1). We found that SET1C and the SET1C762 complex were both associated with Snf2C and Snf2C-AT-hook but not with Snf2C-Bromo (Figure 6B and C), indicating that the AT-hook is a key region for interaction with SET1C. We then delineated the minimal AT-hook region required for interaction with SET1C and found that residues 1461–1547 are essential for binding to SET1C (Figure 6D and E). Collectively, these results indicate that SET1C interacts with the Snf2C-AT-hook region, suggesting that the Set1 and Snf2 complexes cooperate to modify chromatin structure.

**Figure 6—figure supplement 1.**
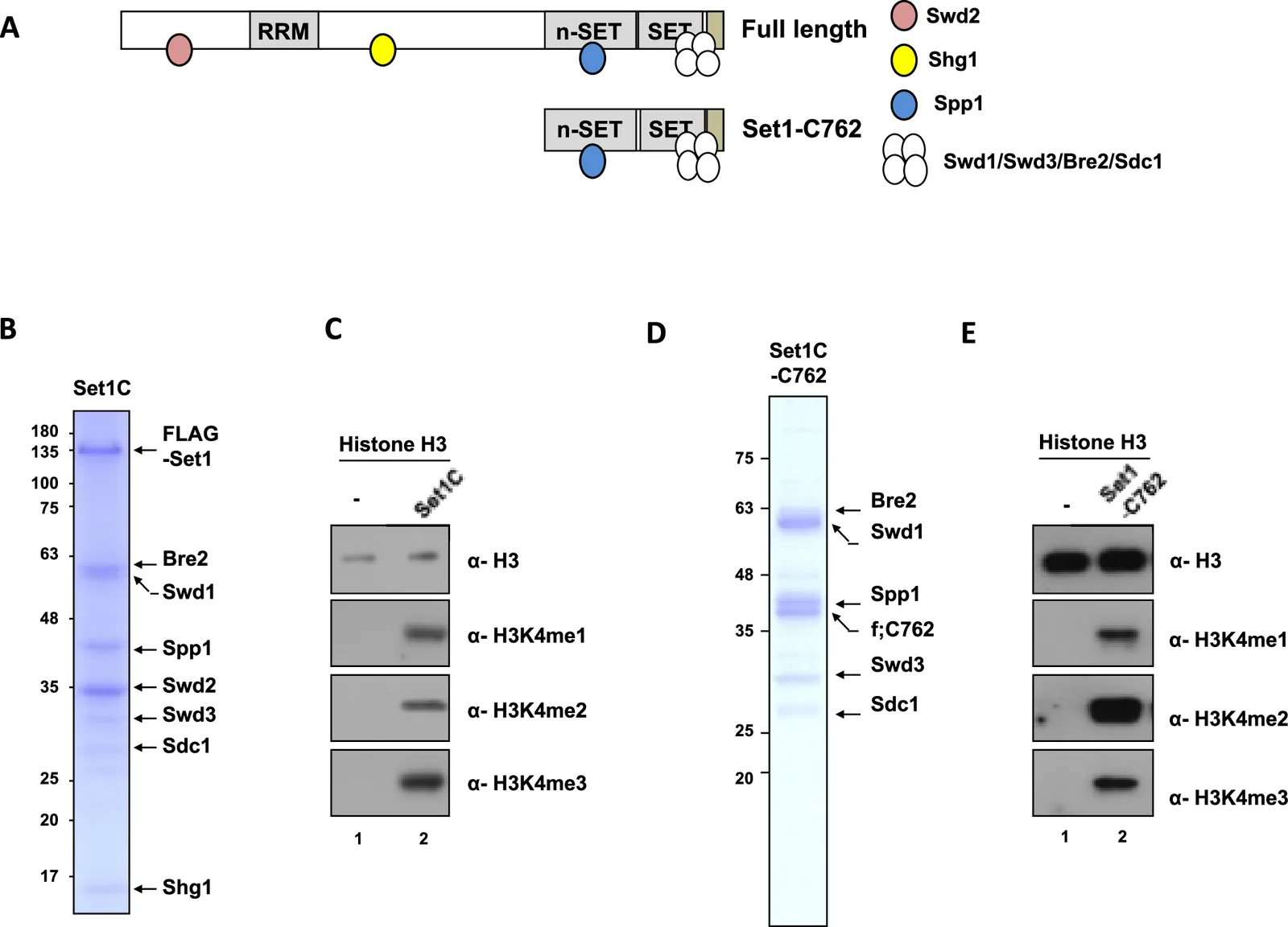
Purification and activity of SET1C and SET1C-C762. (**A**) A schematic diagram of the subunits used for the reconstitution of SET1C and Set1-C762 complexes. (**B**) SDS-PAGE/Coomassie Blue staining of purified SET1C. (**C**) In vitro methyltransferase assay using SET1C and free histone H3. (**D**) SDS-PAGE/Coomassie Blue staining of purified Set1-C762 complex. (**E**) In vitro methyltransferase assay using the Set1-C762 complex and free histone H3. Figure 6—figure supplement 1—source data 1.Source data for the Coomassie blue–stained gels and autoradiographs presented in Figure 6—figure supplement 1. Figure 6—figure supplement 1—source data 2.Uncropped and labelled gel image corresponding to Figure 6—figure supplement 1.

**Figure 7.**
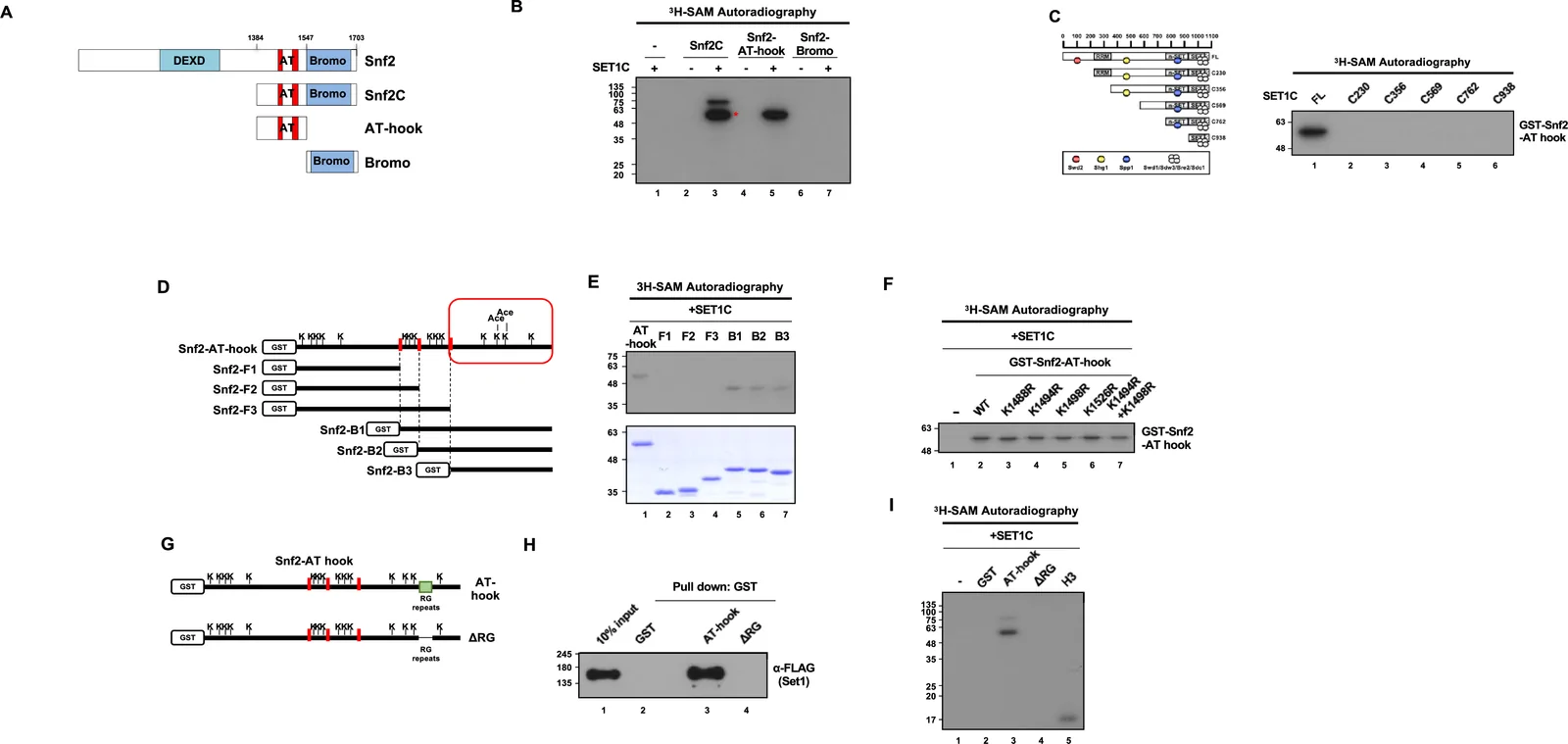
Snf2 is methylated in tandem AT-hook domain by reconstituted Set1C. (**A**) Schematic representation of Snf2 fragments. (**B**) In vitro methyltransferase assay using SET1C incubated with the indicated Snf2 fragments. ^3^H-SAM was used as a methyl donor and methylated proteins were detected by autoradiography. The band marked with a red star is a degradation product of Snf2C. (**C**) Schematic diagram of N-terminal truncated SET1 complexes (left) and in vitro methyltransferase assay with GST-Snf2-AT-hook and truncated SET1 complexes. (**D**) Schematic diagram showing the positions of all lysines in Snf2-AT-hook and the further cleaved fragments of Snf2-AT-hook. The red box indicates the two lysines that are acetylated by Gcn5. (**E**) Coomassie staining of purified Snf2 fragments (lower) and an in vitro methyltransferase assay using these fragments with SET1C (upper). (**F**) In vitro methyltransferase assay by SET1C when each of the four lysines in the C-terminal region of the Snf2-AT-hook is substituted with arginine or when both lysines known to be acetylated by Gcn5 are substituted. (**G**) A schematic diagram showing the position of the RG-repeat region and the design of Snf2-AT-hook with RG-repeat truncation. (**H, I**) GST pull-down assay (**H**) and in vitro methyltransferase assay (**I**) using purified SET1C and GST-Snf2-AT-hook with or without RG-repeats. Figure 7—source data 1.Source data for the Coomassie blue–stained gels, autoradiographs and western blot presented in Figure 7. Figure 7—source data 2.Uncropped and labelled gel image corresponding to Figure 7.

We have shown above that SET1C interacts with the Snf2C-AT-hook region (Figure 6). We thus tested whether the Snf2C-AT-hook could be methylated by Set1C. Set1C and truncated Set1C were reconstituted and affinity-purified from Sf9 cells coinfected with baculoviruses that express FLAG-Set1 (or truncated Set1) and the seven other untagged subunits (Kim et al., 2013). We incubated reconstituted Set1C with the Snf2C, AT-hook, and Bromo fragments in the presence of radioactive S-adenosylmethionine (^3^H-SAM). The results indicate that Set1C methylates in vitro-purified Snf2C and Snf2C-AT-hook, but not Bromo (Figure 7A and B) and that Snf2C-AT-hook methylation requires Set1 FL (Figure 7C). Collectively, these results show that the Snf2C-AT-hook (1384–1547) interacts and is methylated by Set1 FL in vitro. Lys 1494 (K1494) and Lys 1498 (K1498) located between the AT-hook domains of Snf2 were previously shown to be acetylated by Gcn5 (Kim et al., 2010). We individually mutated all the Lys of the Snf2-AT-hook into Arg and tested the methylation of the mutated fragments. None of the substitutions abolished or decreased the methylation of the AT-hook, indicating that the AT-hook could be methylated on multiple sites or on other types of residues (Figure 7—figure supplement 1). We thus sought to identify the minimal region within the Snf2-AT-hook that is methylated by Set1C in vitro. The Snf2-AT-hook domain was divided into six regions that were fused to the GST (Figure 7D). In vitro methylation assays indicated that the Snf2-B1, B2, and B3 were methylated by the reconstituted Set1C (Figure 7E). As the Snf2-B3 fragment that contains the last four lysines (K1488, K1494, K1498, K1526) of the Snf2-AT-hook domain was still methylated, it suggested that one or several of these lysines are methylated by Set1C. We then examined which of these four lysine residues are methylated by Set1C in vitro. To our surprise, mutating all the lysine residues of the Snf3-B3 fragment did not abolish the methylation of Snf3-B3 (Figure 7F). These results led us to think that Set1C could methylate arginine residues, in particular those contained in the RGG motif of the Snf2-B3 fragment. We thus deleted the RG repeats of Snf2-B3 (Figure 7G) and purified the *Snf2-B3∆RG*. We found that deletion of the Snf2-B3 RG repeats abolished the interaction between Snf2-B3 and Set1 and methylation of Snf2-B3 (Figure 7H and I). The fact that both interaction and methylation are lost upon deletion of the RG motif argues in favor of Set1C, and not a potential contaminant from the reconstituted Set1C, being responsible for the methylation of the Snf2-B3 RG repeats. Individual deletion of pairs of arginine residues in the RG motif did not suppress methylation of the Snf2-B3 fragment, suggesting flexibility in Set1C’s ability to methylate Snf2-B3 (Figure 7—figure supplement 2).

**Figure 7—figure supplement 1.**
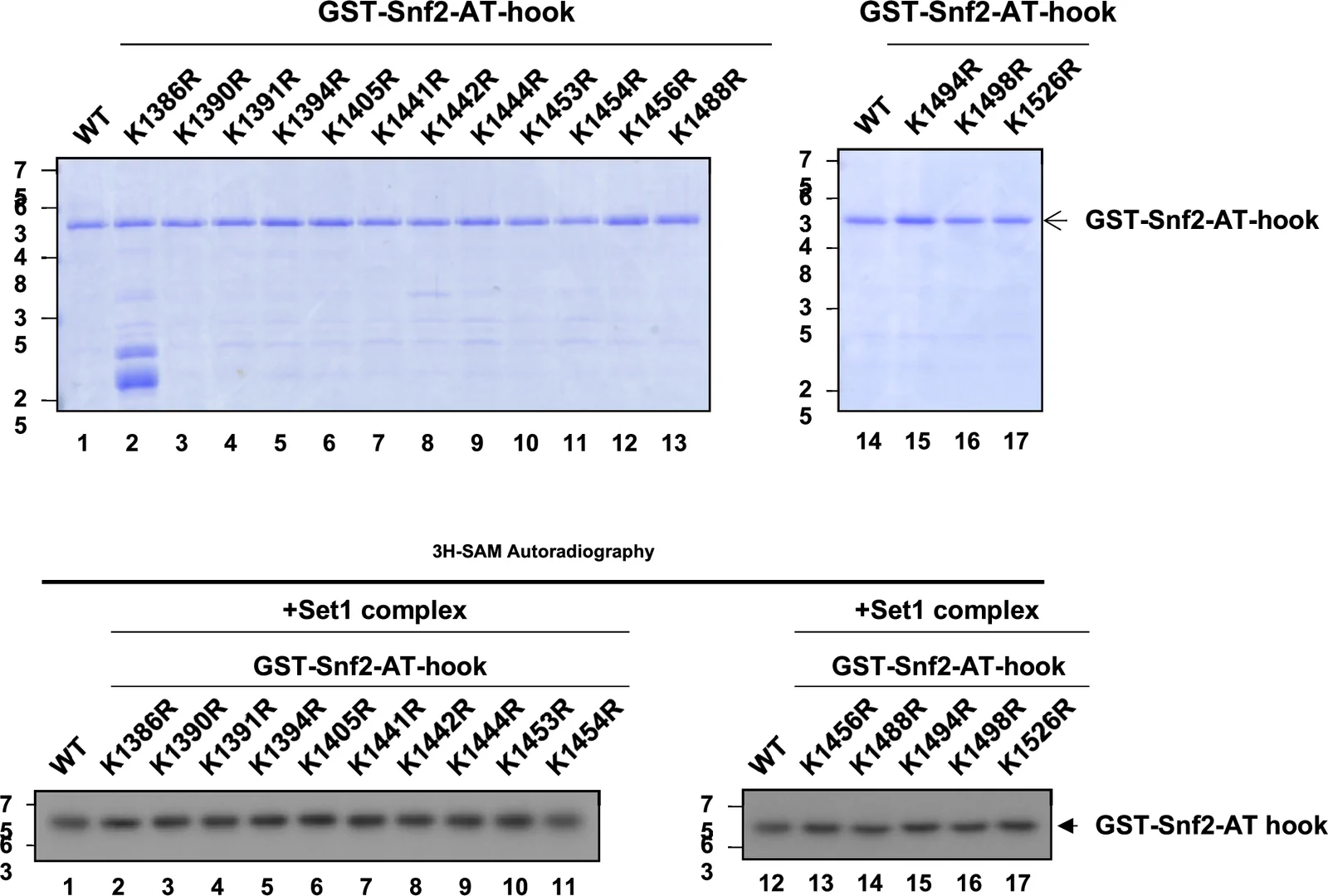
15 lysines of Snf2-AT-hook were replaced with arginine one by one. Purified Snf2-AT-hook wild-type and 15 mutant proteins were subjected to Coomassie staining (upper) and in vitro methyltransferase assay with SET1C (lower). Figure 7—figure supplement 1—source data 1.Source data for the Coomassie blue–stained gels and autoradiographs presented in Figure 7—figure supplement 1. Figure 7—figure supplement 1—source data 2.Uncropped and labelled gel image corresponding to Figure 7—figure supplement 1.

**Figure 7—figure supplement 2.**
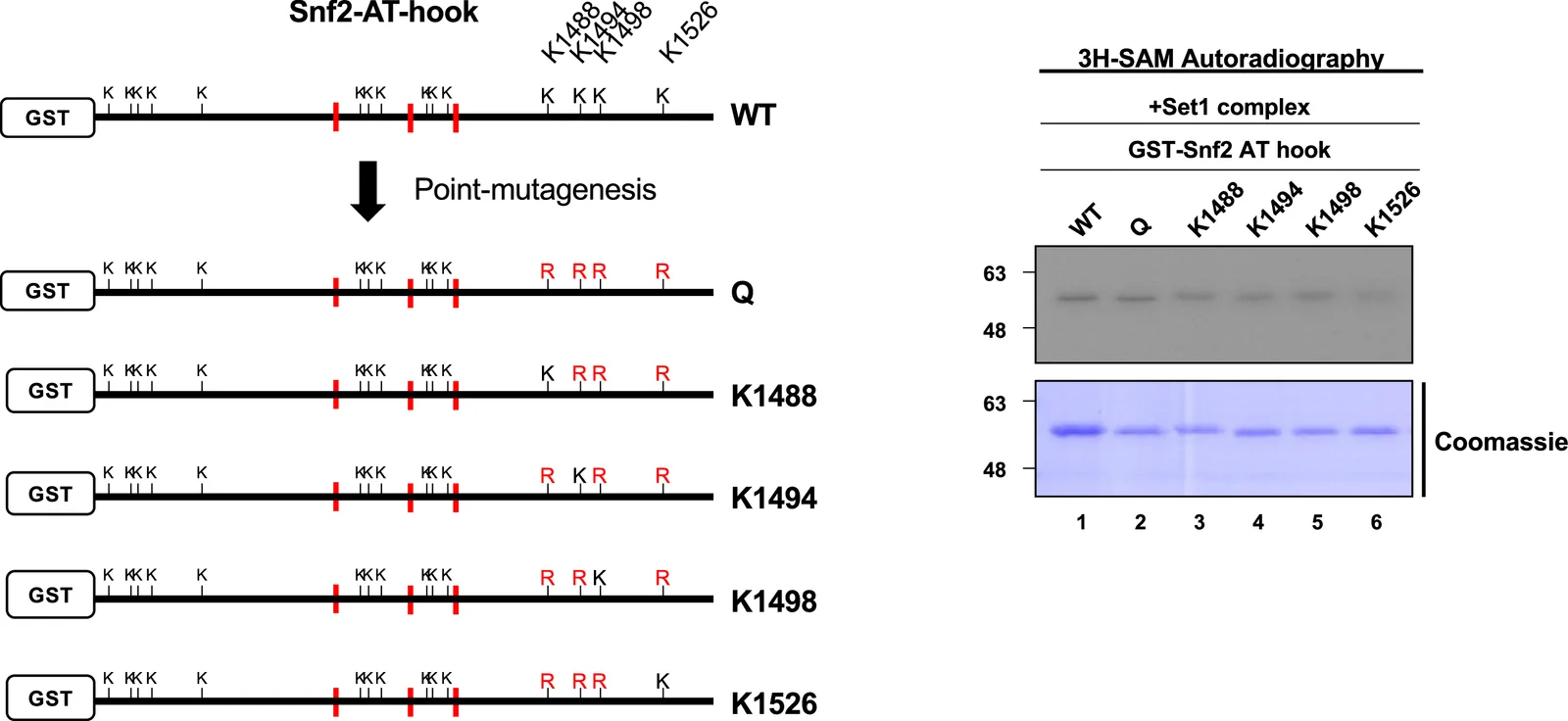
Lysine mutations in the AT-Hook Motifs (K1488, K1494, K1498, and K1526) do not suppress Methylation of the Snf2-B3 Fragment. (**A**) Designed AT-hook mutants with quadra mutation of all four lysines (K1488, K1494, K1498, K1526) or triple mutations among these four lysines. K1494 and K1498 are known to be acetylated by Gcn5. (**B**) In vitro methyltransferase assay using reconstituted SET1C and Snf2-AT-hook mutants. Figure 7—figure supplement 2—source data 1.Source data for the Coomassie blue–stained gels and autoradiographs presented in Figure 7—figure supplement 2. Figure 7—figure supplement 2—source data 2.Uncropped and labelled gel image corresponding to Figure 7—figure supplement 2.

**Figure 8.**
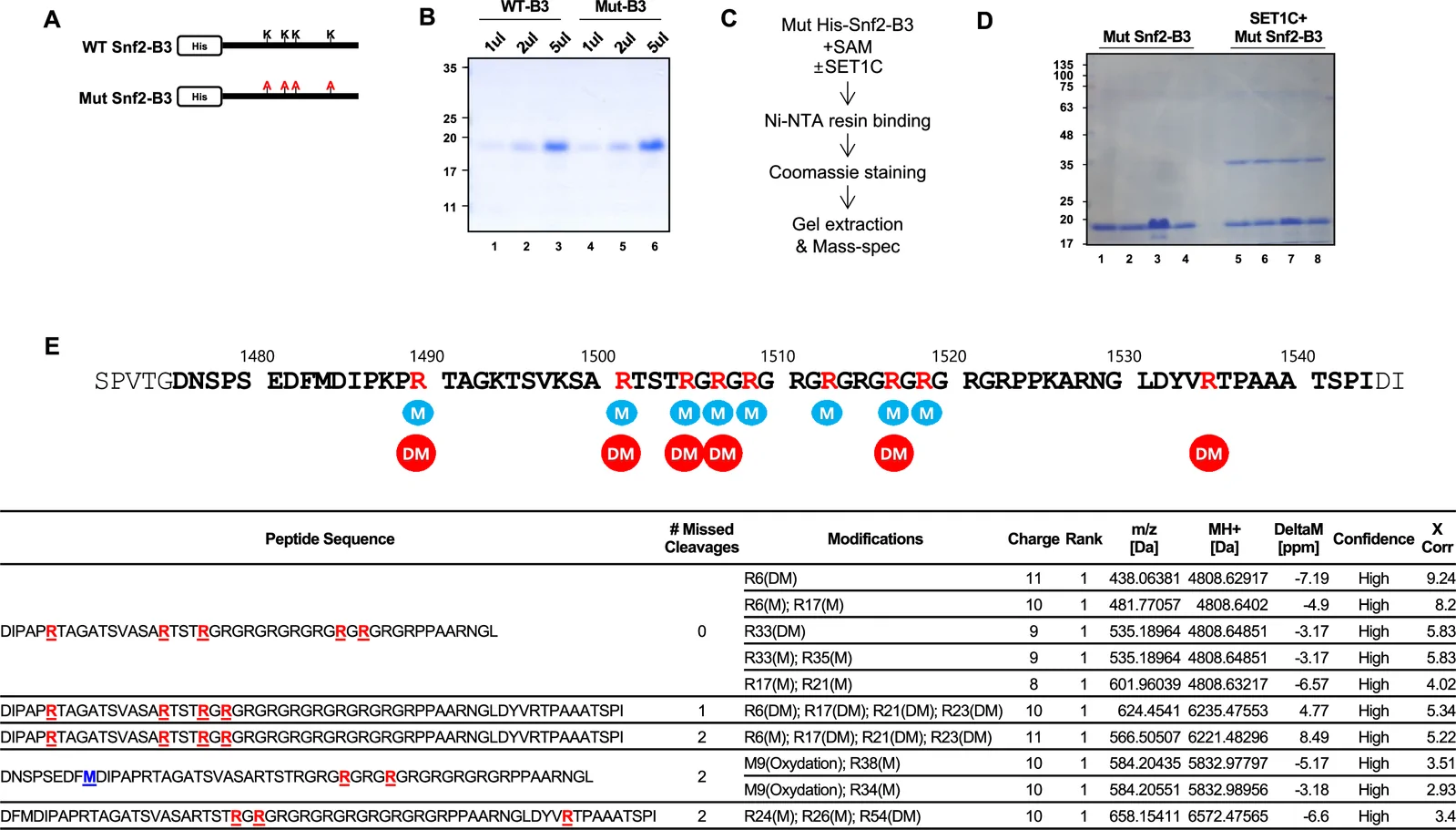
The arginines in the RG-repeat of Snf2 are methylated by reconstituted SET1C. (**A**) A diagram showing the wild-type (WT) Snf2-B3 fragment and the Mut Snf2-B3 with all four lysines substituted with alanine. (**B**) Coomassie staining of purified WT and Mut Snf2-B3. (**C**) Mass spectrometry experiment design to identify the methylation sites of Snf2-B3. (**D**) Coomassie staining of Mut Snf2-B3 after methylation reaction and an additional purification step using Ni-NTA. The band corresponding to Mut Snf2-B3 (~18 KDa) was excised and used for mass spectrometry analysis. The ~37 KDa band observed in lanes 5–8 appears to be a SET1C subunit that binds nonspecifically to Ni-NTA, likely SWD2 based on its size. (**E**) Mass spectrometry analysis result of Snf2-B3 methylation sites revealed that multiple arginines in the RG-repeat were methylated. Amino acid sequence of Snf2 highlighting the arginines methylated by SET1. The sequence of the B3 fragment is shown in bold, and the arginines methylated by SET1 are marked in red. Methylation and demethylation are denoted as M and DM, respectively. Figure 8—source data 1.Source data for the Coomassie blue–stained gels presented in Figure 8. Figure 8—source data 2.Uncropped and labelled gel image corresponding to Figure 8.

To further confirm that Snf2-B3 is methylated on arginine, we incubated a mutant Snf2-B3 (in which lysines were mutated in alanine to limit protease digestion prior to mass spectroscopy analysis) with reconstituted Set1C and SAM and further purified the Snf2-B3 mutant on an Ni-NTA resin (Figure 8A–D). We then analyzed by mass spectroscopy arginine methylation in Snf2-B3. We found that R1490, R1501, R1505, R1507, and R1517 were mono- and di-methylated while R1509, R1513, and R1519 were monomethylated (Figure 8E). We thus confirmed that reconstituted Set1C has the ability to methylate in vitro multiple arginines in the Snf2B3 region.

### Snf2 is methylated in vivo on its ARTSTRGR AT-hook motif in a Set1-dependent way

Our results show that an activity associated with reconstituted Set1C is capable of methylating arginines in the vicinity and in the RG repeats of Snf2C-AT-hook. The fact that only Set1FL is capable of methylating the AT-hook is in favor of this activity being associated with Set1C.

Nevertheless, one cannot formally rule out that methylation of the AT-hook is due to a contaminant from insect cells from which Set1C has been reconstituted.

**Figure 9.**
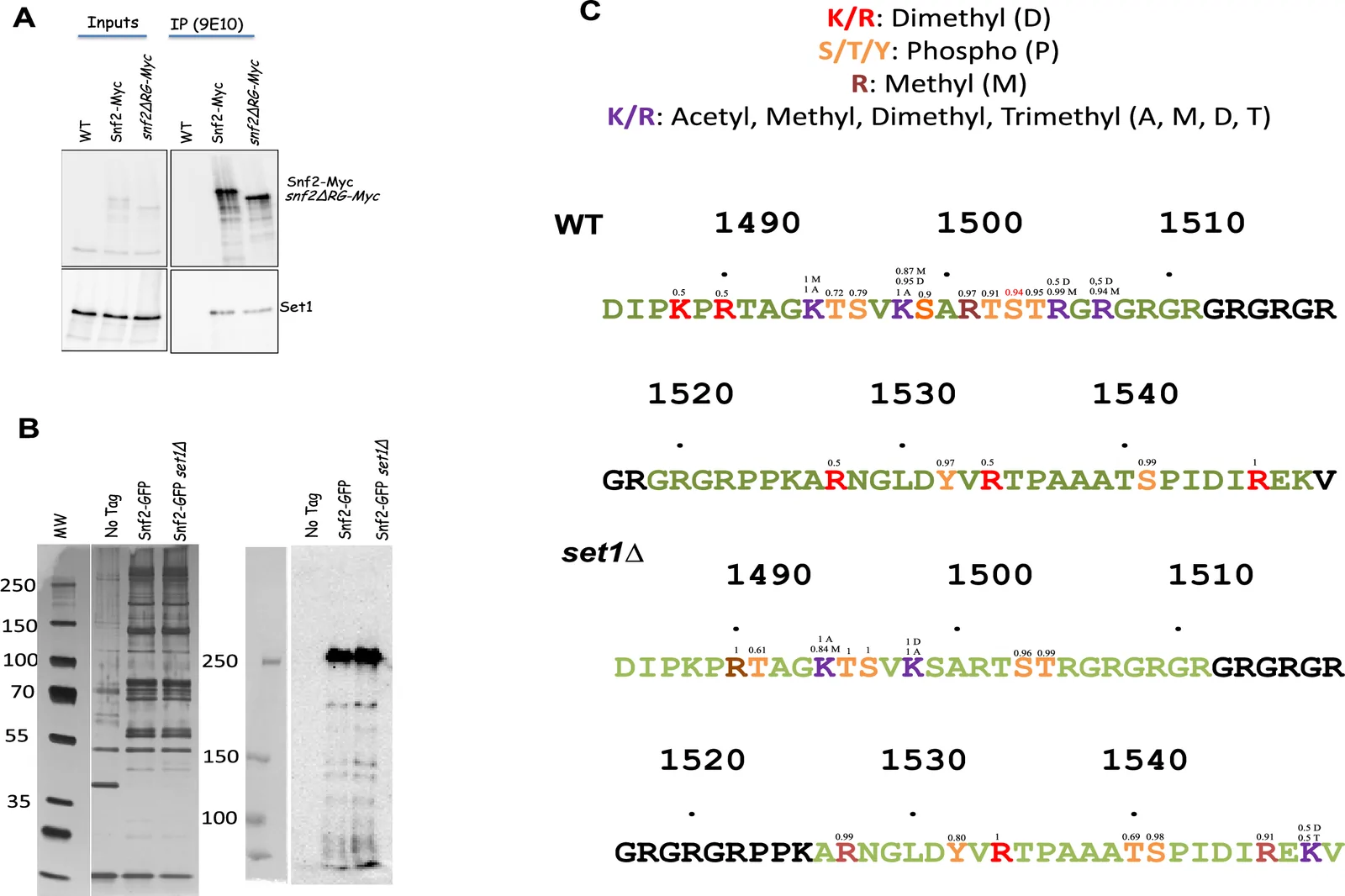
The arginines of the motif ARTSTRGR within the AT-hook are methylated in vivo in a Set1-dependent way. (**A**) Set1 interacts in vivo with Snf2 and Snf2-∆RG. Myc-tagged Snf2 and Snf2∆RG were immunoprecipitated with 9E10 Myc antibodies (see Materials and methods) and revealed with either 9E10 (upper panel) or Set1 antibodies (lower panel). (**B**) Snf2C complex was purified from WT and *set1∆* strains, separated on a 4–12% Bis-Tris gel and silver stained (*left*); the presence of Snf2-GFP is detected by western blotting with anti-GFP antibody (*right*). The area corresponding to Snf2-GFP was excised from the gel and used for mass spectrometry analysis. Peptides flanking the RG repeats (1485–1549) with their post-translational modifications (PTMs) are shown in Figure 9—figure supplement 2. (**C**) Panel C shows a focus on the amino sequence flanking the RG repeats. The positions of residues from D_1485_ to V_1549_ are indicated on the figure. Peptides identified after digestion of Snf2-GFP are indicated in color with their identified PTM indicated by the color code shown in the top of the panel. The small numbers above the amino acids indicate the probability of localization according to the MS2 peaks. It should be noted that for wild-type K1488 and R1490, a peptide with dimethylation is detected, but no discriminating MS2 peak allows us to conclude whether K or R are dimethylated. This is also the case for dimethylation on R1528 and R1535. The results presented represent the observed PTMs from two independent experiments, each containing three replicates (Supplementary file 6).

We thus investigated the interaction between Snf2 and Set1 in vivo and whether Snf2 is methylated by Set1C. We found that Set1 was co-immunoprecipitated with Snf2 tagged with a C-terminal Myc epitope (Figure 9A). In contrast to what we observed in vitro, deletion of the RG motif of Snf2 did not affect the interaction between Snf2 and Set1, which is consistent with the fact that the SID of Snf2 is located upstream of the RG repeats (Figure 9A).

**Figure 9—figure supplement 1.**
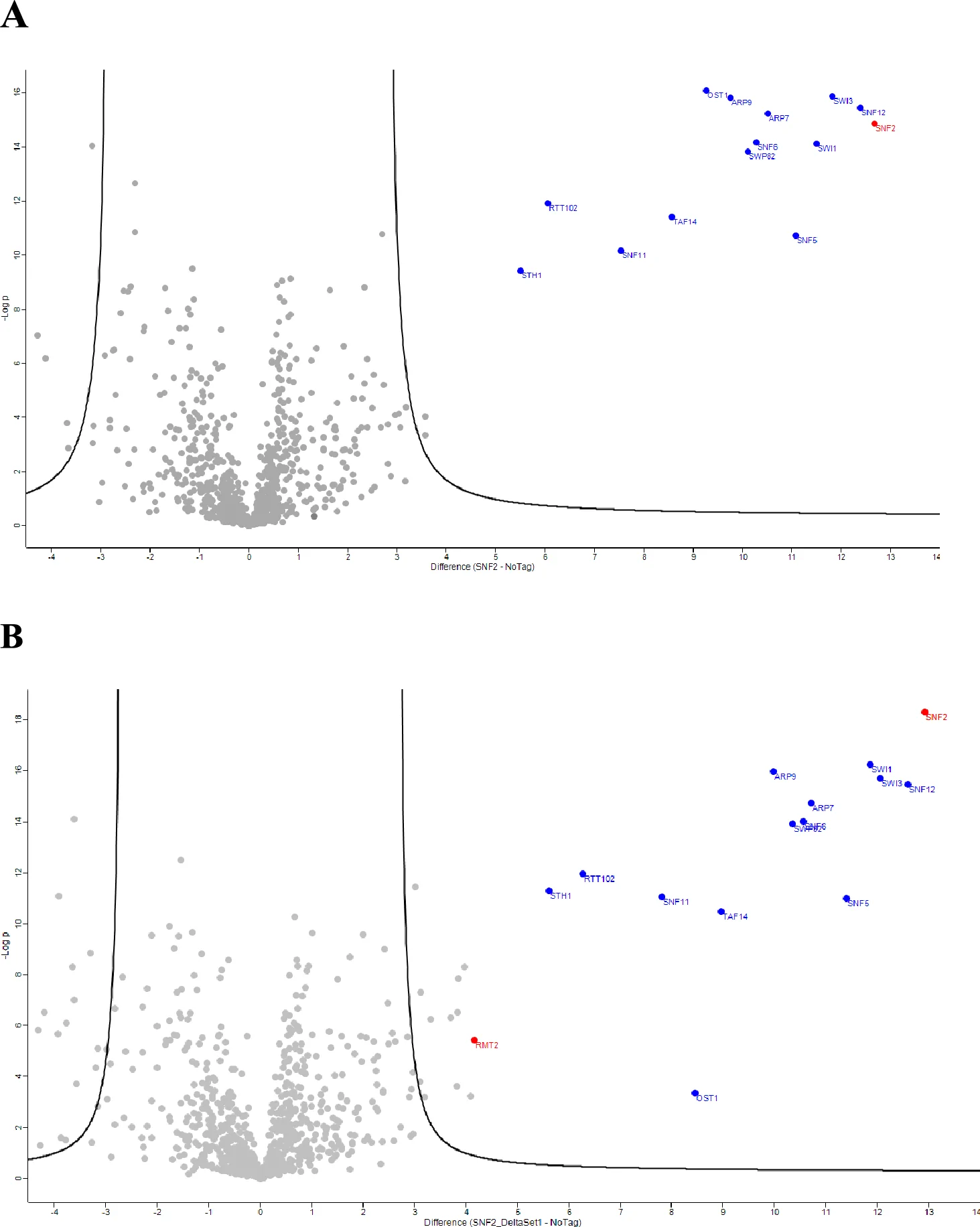
Volcano plot describing the enrichment of proteins in the purifications of Snf2-GFP compared to the control (no tag) (see Supplementary file 5) created by Perseus. (**A**) In a wild-type strain, (**B**) in the *set1∆* strain. The curve represents the significant limit of quantification (S0=5 and false discovery rate [FDR] = 0.01).

**Figure 9—figure supplement 2.**
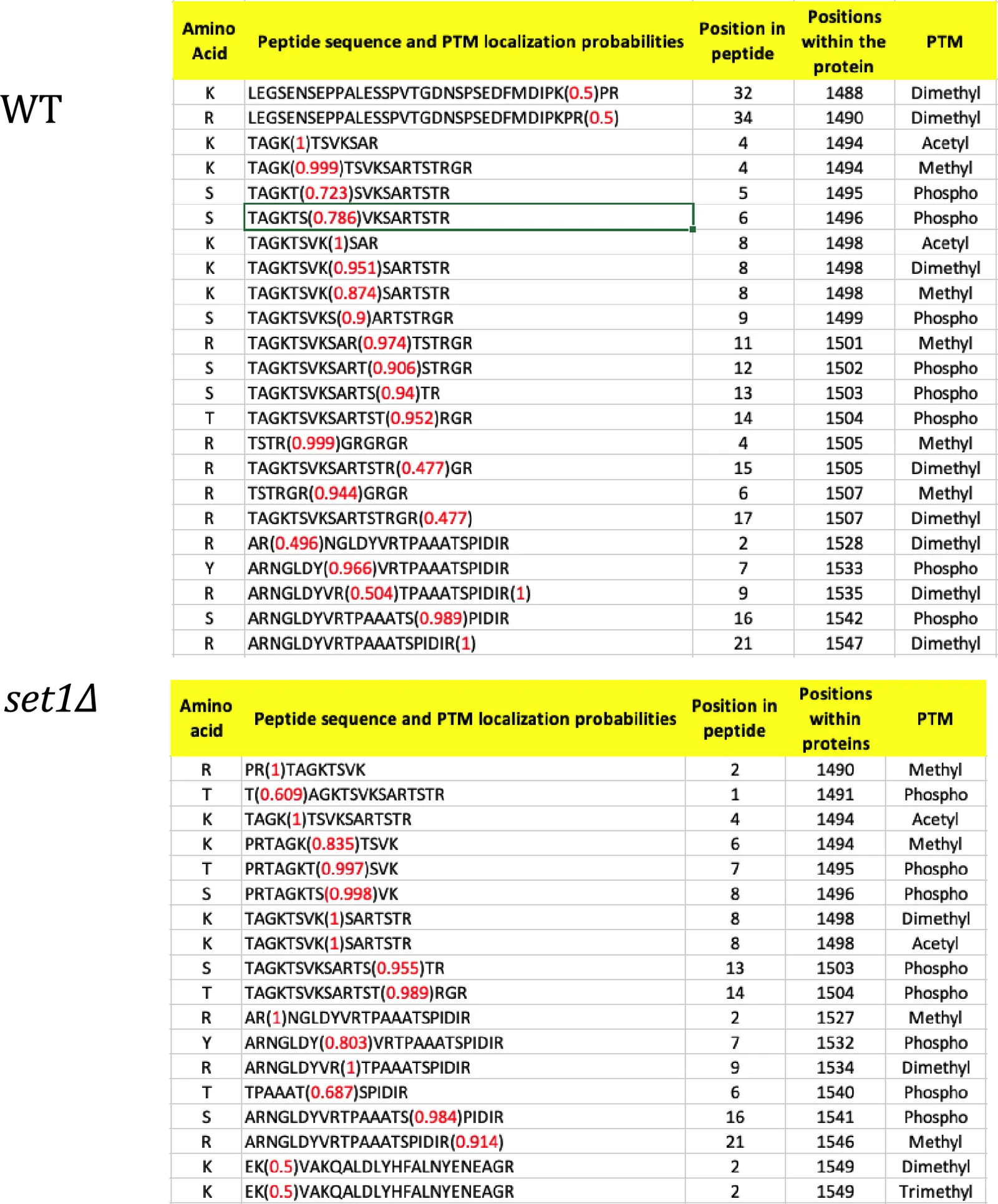
Peptides identified after Snf2-GFP digestion for wild-type (WT) and *set1Δ* are indicated with their identified post-translational modification (PTM). The red numbers indicate the probability of localization according to the MS2 peaks for the preceding amino acid.

To further investigate Snf2 methylation by Set1C in vivo, we purified Snf2-GFP and its subunits with GFP nanobodies in the presence or absence of Set1 (Figure 9B). The protein composition of the complex was determined by mass spectrometry in the presence or absence of Set1 (Figure 9B, Supplementary file 5). We recovered in both cases all the known components of the Snf2 complex (Snf2, Swi1, Swi3, Snf5, Snf12, Swp82, Arp9, Arp7, Snf6, Rtt102, Snf11, Taf14) (Supplementary file 5, Figure 9—figure supplement 1). Interestingly, we found that the arginine methyltransferase Rmt2 was found specifically enriched with the Snf2-GFP complex in *set1∆* cells (Figure 9—figure supplement 1).

We then excised the Snf2-GFP gel band and subjected it to mass spectrometry. We examined the PTMs in the Snf2 (1485–1549) region in either wild-type (WT) or *set1∆* strains (Supplementary file 6). We were unable to purify a peptide containing the RG repeats, but we could identify the peptides flanking the RG motif (Figure 9C). Figure 9C represents the combined results of two independent experiments. In WT cells, we found that the region between K1494 and R1505 was reproducibly subjected to multiple PTMs (Figure 9C, upper panel). K1494 and K1498, which were previously found acetylated by Gcn5 (Kim et al., 2010), were found acetylated; however, both lysines were also found to be methylated (Figure 9C). Of note, H3K4, which is methylated by Set1C, has been reported to be also acetylated by Gcn5 (Guillemette et al., 2011). T1995, S1996, S1999, T1502, S1503, and T1504 were found phosphorylated. Interestingly, R1501 was found mono-methylated while R1505 and R1507 were found di-methylated in the two independent Snf2-GFP purifications. These three arginines belong to an ARTSTRGR motif that lies in the Snf2-B3 fragment. They were also found to be methylated in vitro by the reconstituted Set1C (Figure 8E). We then examined the PTMs of the peptides flanking the RG motif in the *set1∆* strain (Figure 9C, lower panel). Phosphorylation of serines and threonines was not modified in the *set1∆* strain except for T1502. In the *set1∆* strain, K1494 and K1498 remained methylated, indicating that methylation of both lysines does not depend on Set1C. Strikingly, deleting *SET1* abolished the mono-methylation of R1501 and the di-methylation of R1505 and R1507 (Figure 9C, lower panel). The results thus show that SET1C is required for the methylation of the three arginines of the ARTSTRGR motif, in agreement with the in vitro results.

## Discussion

We have exploited the power of Y2H screening technology to establish an extended interaction network for the histone H3K4 methylase complex SET1C. Comprehensive data integration unveiled many potential functions of either the whole complex or individual subunits. This study is partly validated by the fact that 2H interactors were found in known functions of SET1C or putative functions for which the mechanism remains unknown. We have also provided a limited number of validations by confirming the interaction through biochemical approaches. It seems likely that some proteins may interact transiently with Set1 and its subunits, or under specific conditions of stress or nutrient limitations, explaining why these interactors have not previously been found associated with SET1C in biochemical approaches. The most illustrative case for this is the high-affinity interaction between Spp1 and Mer2 that takes place during the first meiotic division, which we had identified from the Spp1 screen and which has been extensively characterized (Acquaviva et al., 2013b). Nevertheless, considering the multitude of interactions identified, it seems unlikely that they all represent direct contacts. The list shown in Supplementary file 2 may contain multiple false positives, and further work will be required to determine whether these interactions occur in a physiological context and what their functional significance may be. Another caveat is that Y2H interactions can be mediated by endogenous proteins that bridge the interactions.

However, we anticipate that this systematic screen will be an invaluable resource for further investigation of the role of SET1C in known and novel processes revealed in this study. Processes discussed here include Set1’s export/import mechanism through the nucleus, interaction with RGG proteins, some of which are targeted by arginine methyltransferase, cooperation of SET1C with chromatin remodeling factors, in particular Snf2C, interaction with splicing factors, notably with Prp8 and Prp22, coupling replication with histone deposition (Mcm2/Spp1), SET1C plasticity regulated by SUMOylation, and Ty element transposition. A recurrent question related to the identification of potential SET1C interactors is whether they could be methylated by SET1C.

We show that Set1C interacts both in vitro and in vivo with Snf2. Strikingly, reconstituted Set1C from insect cells is able to methylate in vitro the three arginines located within the A**R**TST**R**G**R** motif of the AT-hook of Snf2. The in vivo results confirm that Set1 is required for the methylation of these arginines within the A**R**TST**R**G**R** motif. It is interesting to note that this motif resembles the A**R**TKQTA**R** motif of the N-terminal H3. In yeast, H3R2 is mono- and di-methylated, and its asymmetric di-methylation prevents H3K4me3 (Kirmizis et al., 2009; Kirmizis et al., 2007). In contrast, symmetric di-methylation of H3R2 correlates with H3K4me3 and requires Set1 in vivo but not the classical arginine methyltransferases: Hmt1, Hmt2, Rkm2, Rkm3, Rkm4, Hsl7, and Efm1 (Li et al., 2015a; Yuan et al., 2012). It has been proposed that Set1C could be responsible for both H3R2me2s and H3K4me3 or that H3R2me2s deposited by a yet unidentified arginine methyltransferase requires the prior deposition of H3K4me3 (Yuan et al., 2012).

Interestingly, previous tandem affinity protein purification of the mono- and asymmetric arginine methyltransferase Hmt1 (also termed Rmt1) identified 108 proteins associated with it (Jackson et al., 2012), a number of which were shown to be methylated. Snf2 was found to be copurified with Hmt1 in vivo and methylated in vitro by Hmt1 (Jackson et al., 2012), making Hmt1 a potential candidate for methylating Snf2. In vitro, purified Hmt1 catalyzes mono-methylation and asymmetric di-methylation of H3R2; however, deleting *HMT1* does not affect the asymmetric di-methylation of H3R2 in vivo, which suggests that several methyltransferases could act redundantly on H3R2 (Li et al., 2015a). In mammals, H3R2 and H3R8 are asymmetrically di-methylated by PRMT6 (Hamey et al., 2021; Guccione et al., 2007; Hyllus et al., 2007). It has also been reported that PRMT5 can be found associated with hSWI/SNF and has the ability to methylate H3R8 (Pal et al., 2004). We did not identify Rmt1 in our purification but found that the arginine mono-methyl transferase Rmt2 enriched with Snf2-GFP in *set1∆* cells. Rmt2 is a type IV methyltransferase that was reported to methylate the ribosomal protein L12 (Low and Wilkins, 2012; Chern et al., 2002). We could envision the possibility that Set1C could cooperate with distinct protein arginine methyltransferases to promote the mono-methylation of R1501 and the di-methylation of R1505 and R1507 within the A**R**TST**R**G**R** motif. However, it was previously reported that the combined inactivation of Rmt1, Rmt2, and Hsl7 did not affect the mono-methylation of H3R2, weakening this hypothesis (Kirmizis et al., 2009). It is possible that in yeast there is a redundancy of enzymes capable of methylating the Snf2 A**R**TST**R**G**R** motif, but that for each of them their activity on this motif depends on the presence of Set1. Alternatively, Set1C could directly methylate the A**R**TST**R**G**R** motif as discussed for the A**R**TKQTA**R** H3 motif (Yuan et al., 2012). The fact that Set1C interacts with Snf2, Gbp2, Nop1, Nab2, Dbb1 (Figure 2), all of which have RG motifs and are mostly Hmt1 substrates, raises the possibility of a general interplay between methylarginine and methyllysine.

Interestingly, a very recent article shows that PRMT1 binds to the N-terminal region of MLL2 and methylates multiple arginine residues within its RGG/RG motifs (An et al., 2025).

## Materials and methods

### Strain construction

All strains and plasmids used in this study are listed in Supplementary file 1. To obtain gene deletions and expression of tagged proteins, we amplified by PCR a disruption or a tagging cassette containing the appropriate marker as described (Janke et al., 2004).

### Y2H analysis

Y2H screening was performed by Hybrigenics Services, S.A.S., Evry, France. The coding sequence for Set1-FL, Set1 1–754, Set1 754–1081, and Bre2 were cloned into pB66 (GAL4-bait) as C-terminal fusions to the Gal4 DNA-binding domain while those of Swd1, Swd3, Sdc1, Spp1, and Shg1 were cloned into pB27 as a C-terminal fusion to LexA (LEXA-bait). *SWD2* was cloned into pB43 as an N-terminal fusion (SWD2-GAL4). Individual bait cloning was performed by using specific primers for PCR for every bait. Every PCR fragment subcloned as bait is entirely sequenced to avoid eventual mismatches in the coding sequence. The constructs were used as baits to screen a genomic *S. cerevisiae* library constructed into pP6 based on the pGADGH plasmid (Wilson et al., 1993). pB6, pB66, and pB43 are derived from the original pAS2Δ vector (Fromont-Racine et al., 1997). The library was submitted to very strict quality controls. Each protein is represented by several fragments (domains), and the library has been screened with a set of six reference baits before any other bait proteins are screened.

The Gal4 constructs were screened using a mating approach with YHGX13 (Y187 *ade2-101::loxP-kanMX-loxP, MATalpha*) and CG1945 (*MATa*) yeast strains. The LexA constructs were screened using a mating approach with YHGX13 (Y187 *ade2-101::loxP-kanMX-loxP, mat alpha) and L40∆Gal4* (*MATa*) yeast strains as previously described (Fromont-Racine et al., 1997). Positive clones were selected on a medium lacking tryptophan, leucine, and histidine and supplemented with 3-aminotriazole (3AT) if necessary to handle bait auto-activation. The prey fragments of the positive clones were amplified by PCR and sequenced at their 5’ and 3’ junctions. The resulting sequences were used to identify the corresponding interacting proteins in the GenBank database (NCBI) using a fully automated procedure.

A confidence score (PBS, for Predicted Biological Score) was attributed to each interaction as previously described (Formstecher et al., 2005). The confidence score relies on two different levels of analysis. First, a local score takes into account the redundancy and independency of prey fragments, as well as the distribution of reading frames and stop codons in overlapping fragments. Second, a global score takes into account the interactions found in all the screens performed at Hybrigenics using the same library. This global score represents the probability of an interaction being nonspecific. For practical use, the scores were divided into four categories, from A (highest confidence) to D (lowest confidence). A fifth category (E) specifically flags interactions involving highly connected prey domains previously found several times in screens performed on libraries derived from the same organism. Finally, several of these highly connected domains have been confirmed as false positives of the technique. The PBS scores have been shown to positively correlate with the biological significance of interactions. When possible, the bait interacting domain of each prey is provided. The PBS scores have been shown to positively correlate with the biological significance of interactions (Rain et al., 2001). e-Values for the interactions are available in the Hybrigenics database.

### Protein expression and protein interaction assays

Protein expression was done in *Escherichia coli* BL21 cells and purification of GST-fusion proteins were as described (Dichtl et al., 2002). MBP-fusion proteins were purified according to the manufacturer’s instructions (New England BioLabs, Beverly, MA, USA). For His-tagged proteins expressed in bacteria, cDNAs were inserted into the pET28 vector (Novagen), expressed in *E. coli*, and purified using Ni-NTA beads (QIAGEN) following the previously described procedure (Kim and Roeder, 2011). GST pull-down assays were done as described (Dichtl et al., 2002).

A baculovirus expression system was used to express and reconstitute Set1 complexes containing either FLAG-Set1 or FLAG-Set1-C762. cDNAs were subcloned into pFASTBAC1, with or without an epitope tag, and baculoviruses were produced following the manufacturer’s instructions (Gibco-Invitrogen). Sf9 cells were infected with various combinations of baculoviruses, and the complexes were purified by affinity chromatography using M2 agarose, as previously described (Kim and Roeder, 2011).

### TAP-Nis1 affinity purification and mass spectrometry analysis

TAP-Nis1 and control cells (W303a) were grown in YPD, harvested in logarithmic phase (OD_600_ 0.6–0.85), and cryo-lysed as previously described (Trahan and Oeffinger, 2022). Affinity purification was performed in RNP100 (20 mM HEPES-KOH pH 7.4, 100 mM NH_4_OAc, 0.5% Triton X-100, 0.1% Tween 20, 1:100 solution P, 1:5000 antifoam A, 100 mM NaCl) as described in Trahan and Oeffinger, 2022. Following washes, the samples were on-bead trypsin-digested in a volume of 50 µl (f.c. 20 μg/mL trypsin (Sigma, proteomics grade) in 20 mM Tris-HCl (pH 8.0), 37°C, 900 rpm, 16–20 hr; stopped with 2% formic acid) and analyzed by tandem mass spectrometry as described in Trahan and Oeffinger, 2022, using a 70 min gradient on an LTQ Orbitrap Velos, a hybrid mass spectrometer (Thermo Fisher Scientific) in data-dependent mode. Data were processed with Thermo Excalibur to generate a raw file. Mascot search server (53 version 2.3.02) was used with a parent tolerance of 10 ppm for precursor ions, 0.52 Da for fragments, and only considering one possible missed cleavage, as well as a mass change of +16 for methionine oxidations in the mass calculation. Data were searched against *S. cerevisiae* NCBI database and analyzed in Scaffold (version 3.6.4). The threshold and false discovery rates (FDRs) were set to 80% and 0.37%, respectively, for peptides, and to 95% (one peptide minimum) and 1.9% for proteins, respectively. Exclusive spectrum counts were used for analysis, and for each prey, the highest values obtained in the controls were removed from those of the samples during analysis.

### In vitro methylation

In vitro methylation reactions were done essentially as described (Roguev et al., 2001). 30 ml reactions typically contained 2–4 ml of partially purified SET1C complex, 2–4 mg substrate (either peptide, or core histone mixture, or recombinant H3), and 2 ml S-adenosyl (methyl-^3^H) methionine in MTA buffer (50 mM Tris 8.5, 20 mM KCl, 10 mM MgCl_2_, 250 mM sucrose). Reactions with core histones and recombinant H3 were resolved on 4–20% NuPAGE gels and subjected to fluorography. For Snf2 methylation assays, reaction mixtures containing purified SET1C (with 30 ng of the Bre2 subunit) and 200 ng of Snf2 fragments in 20 μl of reaction buffer (25 mM HEPES [pH 7.6], 50 mM KCl, 5 mM MgCl_2_, 0.1 mM EDTA, and 10% glycerol) were supplemented with 1 μCi of S-adenosyl (methyl-^3^H) methionine (PerkinElmer) and incubated at 30°C for 1 hr. Proteins were resolved by SDS-PAGE and subjected to fluorography. For fluorography, gels were fixed for 30 min in 40% methanol, 10% acetic acid, treated with EN^3^HANCE solution (Perkin Elmer) for 60 min, washed in cold dH_2_O for 30 min, dried, and exposed for 5–14 days to photographic film.

### Mass spectrometry-based identification of Arg methylation in Snf2-B3

Mutant Snf2-B3 underwent a methylation reaction with SET1C and SAM, followed by purification via Ni-NTA affinity chromatography and separation by SDS-PAGE. The gel bands corresponding to the mutant Snf2-B3 were excised and subjected to in-gel digestion with AspN enzyme, followed by peptide extraction. The resulting peptide fractions were analyzed using an Easy-nLC 1200 coupled to an Orbitrap Fusion Lumos mass spectrometer (Thermo Fisher Scientific, MA, USA) at the Korea Basic Science Institute (Ochang). Peptides were separated on a C18 column using a 150 min gradient, and data were acquired in data-dependent acquisition (DDA) mode. Full MS scans were acquired in the Orbitrap at a resolution of 60,000 over an m/z range of 350–2000. Precursors for MS/MS analysis were selected for higher-energy collisional dissociation (HCD) fragmentation at a normalized collision energy of 30%. Raw MS data were processed using Proteome Discoverer with the SEQUEST search engine. The data were searched against the sequence of Snf2-B3 with a precursor mass tolerance of 10 ppm and a fragment mass tolerance of 0.02 Da. AspN was set as the digestion enzyme, allowing for up to two missed cleavages. Methylation, dimethylation, and methionine oxidation were specified as variable modifications. The FDR was set to 1% at the peptide level.

### Y2H interaction of Set1 fragments with selected preys

The Set1 fragments were amplified from the pB66-Set1-FL plasmid and cloned into the SfiI site of pB66. The selected preys (Snf2, Prp8, and Prp22) into p6 (Hybrigenics) were extracted from the screen. Plasmid pB66 contains the Gal4 DNA-binding domain and the *TRP1* marker, while pP6 expresses the Gal4 activating domain and the *LEU2* marker. TOTO cells were transformed with the different pB66-Set1-Fragments and the pP6-interactors and incubated 3 days at 30°C on SD-LEU-TRP. The transformant colonies were then streaked on SD-LEU-TRP-HIS containing either 5 mM or 20 mM of 3AT. To visualize interaction between Set1 fragments and the interactors, yeast cells were incubated 2 days at 30°C and cell growth was examined.

### AlphaFold model

AlphaFold models were generated as described in Abramson et al., 2024.

### Co-immunoprecipitation of Prp22-FLAG with Myc-Set1

Co-immunoprecipitation experiment was performed in W303 expressing chromosomally encoded Myc-Set1 (Dehé et al., 2006a) and Prp22AID-FLAG (Mendoza-Ochoa et al., 2019). 250 ml of culture at an OD_600_ of 0.8 was harvested by centrifugation at 1000 × *g* and washed twice in ice-cold 1× PBS. The cell pellet was resuspended in 900 µl lysis buffer (50 mM Tris-HCl pH 7.5, 2 mM Mg2Cl_2_, 150 mM NaCl, 0.2% NP-40, and one complete EDTA-free proteinase inhibitor tablet [Roche #11836145001]) and 400 µl zirconia beads. Cells were lysed using a Mini-Beadbeater-24 (BioSpec Products) twice at 2000 rpm for 2 min followed by 2 min on ice. The sample was centrifuged at 1000 × *g* for 2 min; the supernatant was collected and additionally centrifuged at 20,000 × *g* for 30 min at 4° and used for immunoprecipitation. The concentration of protein was measured using the Bradford assay, and 1 mg of protein was used per immunoprecipitation. Prior to immunoprecipitation, extract was pre-cleared by adding ½ volume unconjugated Protein A/G Dynabeads (Life Technologies ##10001D/10003D). 50 µl Protein A/G Dynabeads conjugated to antibody were incubated with the appropriate volume of extract on a rotating wheel overnight at 4°C. The next day, beads were washed eight times in lysis buffer (non-bound fraction kept for analysis). 20 µl of loading buffer was added to the beads, input, and non-bound samples, which were boiled for 10 min before loading on a NuPAGE 4–12% Bis-Tris gel (Invitrogen), and western blotting was performed.

### Set1 SUMOylation analysis

Cells were transformed with a plasmid encoding 6His-SUMO under the *CUP1* promoter (YEp352-6His-SUMO) or the corresponding empty vector (Niño et al., 2016). The transformed cells were grown on selective medium and stimulated overnight with 0.1 mM CuSO_4_. 200 OD_600_ of cells were collected and lysed with glass beads in a 20% TCA solution; the final TCA concentration was adjusted to 12%. Cell lysates were incubated at 4°C for 45 min, and precipitated proteins were collected by centrifugation. Proteins were resuspended in loading buffer (6 M guanidinium-HCl, 100 mM KH_2_PO_4_, 20 mM Tris-HCl, pH 8.0, 100 mM NaCl, 0,1% Triton X-100, and 10 mM imidazole). 6His-SUMOylated proteins were purified using Ni-NTA agarose beads (QIAGEN), pre-equilibrated with loading buffer, and incubated for 1 hr at room temperature (RT). After incubation, the beads were collected by centrifugation (3000 rpm, 2 min) and washed twice with wash buffer (8 M urea, 100 mM Na_2_HPO_4_/NaH_2_PO_4_ pH 6.4, 10 mM Tris-HCl, pH 6.4, 10 mM imidazole, 10 mM β-mercaptoethanol, and 0.1% Triton X-100). 6His-SUMOylated proteins were eluted with 50 ml 2× Laemmli buffer (95°C, 5 min). The proteins in the eluted fraction were analyzed by western blot using anti-MYC (for endogenous Myc-Set1), anti-GAL4 (for GBD-Set1-full length and fragments), and polyclonal rabbit anti-Smt3.

### Interaction of Snf2-AT-hook with SET1C and SET1C-762

GST-tagged Snf2 fragments (final concentration 250 nM) were combined with either the full-length SET1C or C762SET1C (final concentration 25 nM), along with 15 µl of glutathione-Sepharose 4B resin and BSA (final concentration 0.2 mg/ml) in a binding buffer containing 20 mM Tris-Cl (pH 7.9), 150 mM NaCl, 0.2 mM EDTA, 20% glycerol, 0.1% NP-40, and 1 mM PMSF, making a total volume of 300 µl. The mixtures were rotated at 4°C for 3 hr, followed by four washes with the binding buffer. The proteins bound to the resin were then eluted, separated by SDS-PAGE, and analyzed by immunoblotting.

### Co-immunoprecipitation of Snf2-Myc and Snf2∆RG-Myc with Set1

WT cells or expressing *Snf2-Myc* and *Snf2∆RG-Myc* (200 ml) were grown at 30°C in YPD to an OD_600_=0.8. Cells were collected by centrifugation, washed once with 10 mM Tris-HCl, pH 8.0, and snap-frozen in liquid nitrogen. Cells were lysed using Retsch with the following parameters: two times 2 min at 30 m/s. The powder was resuspended in 3 ml of TMG 50 (10 mM Tris-HCl, pH 8.0, 0,1 mM MgCl_2_, 10% [vol/vol] glycerol, 50 mM NaCl, 0.1 mM EDTA, 0.1 mM DTT) containing protease inhibitors, 10 mM MG-132 and 1 mM PMSF. Cell lysates were clarified by centrifugation (13,300 rpm, 15 min, 4°C), and 1.4 ml of supernatant were recovered. Protein concentration was determined using nanodrop, and samples were adjusted to the same concentration by adding lysis buffer. Immunoprecipitation was performed overnight with 5 ml of 9E10 antibody (Santa Cruz Biotechnology) followed by an incubation for 3 hr with 25 ml of pre-equilibrated protein-G dynabeads (Invitrogen). Immunoprecipitations were washed 3× with TMG 50 buffer and eluted using 25 ml of 1× Laemmli loading buffer. Samples were resolved on a 7.5% acrylamide gel, transferred onto a nitrocellulose membrane, and revealed with either anti-Myc 9E10 (Santa Cruz Biotechnology) or anti-Set1 antibodies.

### Snf2-GFP complex purification

800 ml of *Snf2-GFP* and *Snf2-GFP set1∆* cells were grown at 30°C in YPD to an OD_600_=0.8. Cell pellets were treated as described above except that supernatants were incubated with 25 ml of GFP nanobody-coated Dynabeads (gift from M Modesti, CRCM Marseille) for 250 min, washed 3× with TMG 50 buffer, and eluted in 30 ml of 2× Laemmli loading buffer containing 50 mM DTT. For western blotting, 1 ml was resolved on a NuPAGE 4–12% Bis-Tris Gel (Invitrogen), transferred onto a nitrocellulose membrane, and revealed using an anti-GFP antibody (A-11122-Invitrogen). For the silver staining, 2,5 ml were resolved on a NuPAGE 4–12% Bis-Tris Gel (Invitrogen) and stained using Pierce Silver Stain kit (Thermo Scientific).

### Mass spectrometry-based identification of PTMs in Snf2-GFP

Purified Snf2-GFP (n=3, biological replicates) from WT and *set1∆* strains were loaded on NuPAGE 4–12% Bis-Tris acrylamide gels according to the manufacturer’s instructions (Life Technologies). Running of protein was stopped as soon as proteins stacked in a single band. Protein containing bands were stained with Imperial Blue (Pierce), cut from the gel, and digested with high-sequencing grade trypsin (Promega) before mass spectrometry analysis. Briefly, gel pieces were washed and destained using a few steps of 100 mM NH_4_HCO_3_. Destained gel pieces were shrunk with 100 mM ammonium bicarbonate in 50% acetonitrile and dried at RT. Protein spots were then rehydrated using 10 mM DTT in 25 mM ammonium bicarbonate pH 8.0 for 45 min at 56°C. This solution was replaced by 55 mM iodoacetamide in 25 mM ammonium bicarbonate pH 8.0, and the gel pieces were incubated for 30 min at RT in the dark. They were then washed twice in 25 mM ammonium bicarbonate and finally shrunk by incubation for 5 min with 25 mM ammonium bicarbonate in 50% acetonitrile. The resulting alkylated gel pieces were dried at RT. The dried gel pieces were reswollen by incubation in 25 mM ammonium bicarbonate pH 8.0 supplemented with 12.5 ng/µl trypsin (Promega) for 1 hr at 4°C and then incubated overnight at 37°C. Peptides were harvested by collecting the initial digestion solution and carrying out two extractions: first in 5% formic acid and then in 5% formic acid in 60% acetonitrile. Pooled extracts were dried down in a centrifugal vacuum system. Samples were reconstituted in 0.1% TFA, 4% acetonitrile before mass spectrometry using an Orbitrap Fusion Lumos Tribrid Mass Spectrometer (Thermo Fisher Scientific, San Jose, CA, USA) online with an Ultimate 3000RSLCnano chromatography system (Thermo Fisher Scientific, Sunnyvale, CA, USA). Peptides were separated at 40°C using a two-step linear gradient (4–20% acetonitrile/H_2_O; 0.1% formic acid for 110 min and 20‐32% acetonitrile/H_2_O; 0.1% formic acid for 10 min). An EASY‐Spray nanosource was used for peptide ionization (2200 V, 275°C). MS was conducted using a DDA. The Orbitrap Lumos was used in data-dependent mode to switch consistently between MS and MS/MS. Time between Masters Scans was set to 3 s. MS spectra were acquired with the Orbitrap in the range of m/z 400–1600 at an FWHM resolution of 120,000 measured at 400 m/z. AGC target was set at 4.0e5 with a 50 ms maximum injection time. For internal mass calibration, the 445.120025 ion was used as lock mass. The more abundant precursor ions were selected, and collision-induced dissociation fragmentation was performed in the ion trap to have maximum sensitivity and yield a maximum amount of MS/MS data. The number of precursor ions was automatically defined along the run in 3 s windows using the ‘Inject Ions for All Available parallelizable time option’ with a maximum injection time of 300 ms. The signal threshold for an MS/MS event was set to 5000 counts. Charge state screening was enabled to exclude precursors with 0 and 1 charge states. Dynamic exclusion was enabled with a repeat count of 1 and duration of 60 s.

For identification of PTMs, the region corresponding to Snf2-GFP was excised from the gel and subjected to enzymatic digestion under the same conditions as above. Raw mass spectrometry files were analyzed using MaxQuant software (version 1.6.3.4) as above with the exception of the maximum number of missed cleavages set to 5, the FDR at the peptide and protein levels was set to 5%, with the following variable modifications activated: lysine acetylation (+42.0106); serine, threonine, and tyrosine phosphorylation (+79.966); lysine and arginine methylation (+14.0157), dimethylation (+28.0313), and trimethylation (+42.0470).

### Data processing protocol

Relative intensity-based label-free quantification (LFQ) was processed using the MaxLFQ algorithm from the freely available MaxQuant computational proteomics platform, version 1.6.2.1 (Cox et al., 2014; Cox and Mann, 2008). The acquired raw LC Orbitrap MS data were first processed using the integrated Andromeda search engine. Spectra were searched against the *Saccharomyces cerevisiae* (organism ID 4932) extracted from UniProt containing 7904 entries. The following parameters were used for searches: (i) trypsin allowing cleavage before proline; (ii) two missed cleavages were allowed; (iii) cysteine carbamidomethylation (+57.02146) as a fixed modification and methionine oxidation (+15.99491) and N-terminal acetylation (+42.0106) as variable modifications. The match between runs option was enabled. The false discovery rate (FDR) at the peptide and protein levels was set to 1% and determined by searching a reverse database. For protein grouping, all proteins that cannot be distinguished based on their identified peptides were assembled into a single entry according to the MaxQuant rules. The statistical analysis was done with the Perseus program (version 1.6.1.3) (Tyanova and Cox, 2018) from the MaxQuant environment (https://maxquant.org/). Quantifiable proteins were defined as those detected in above 70% of samples in one condition or more. Protein LFQ normalized intensities were base 2 logarithmized to obtain a normal distribution. Missing values were replaced using data imputation by randomly selecting from a normal distribution centered on the lower edge of the intensity values that simulates signals of low abundant proteins using default parameters (a downshift of 1.8 standard deviations and a width of 0.3 of the original distribution). The protein composition of the complex was determined using a two-sample t-test using permutation-based FDR-controlled at 0.01 and employing 250 permutations and a scaling factor s0 with a value of 3. All proteins passing these criteria with a positive fold change are considered to be the potential interactome of Snf2.

### Antibodies

For IP: Mouse anti-FLAG (Sigma M2 #F1804); 10 μl/IP with Dynabeads and Protein G.

For westerns – primary antibodies: Mouse anti-c-MYC (Santa Cruz #SC-40x) 1:1000; Rat anti-FLAG (Agilent #200474) 1:1000; Mouse anti-Gal4 Binding Domain (Euromedex) 1:5000; Rabbit anti-Smt3 is a gift from B Palancade (Institut Jacques Monod, Paris). Anti-Bre2 is a gift from Peter Nagy. The rabbit anti-Spp1 antibody was developed in V Géli’s lab. The anti-Spp1 antibody is showing significant cross-reactivity with other cellular proteins but did allow the identification of Spp1 in purified and partially purified fractions. Secondary antibodies: Goat anti-mouse IRDye680RD (LI-COR #926-68070) 1:10,000, Goat anti-rat IRDye800RD (LI-COR #926-32219) 1:10,000.

## Figures and Tables

**Appendix 1—figure 1.**
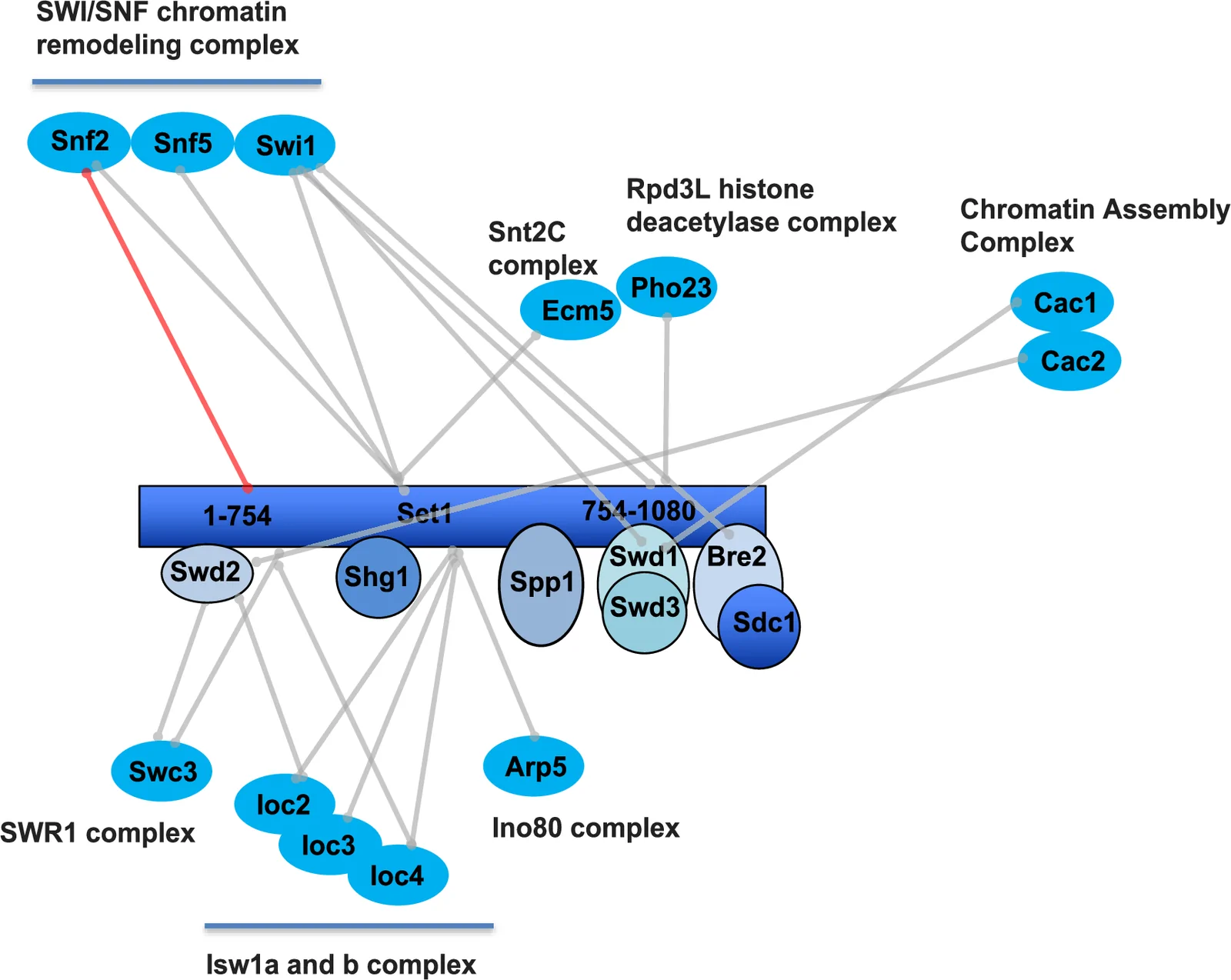
Chromatin regulators identified in all the two-hybrid screens. The lines indicate the individual proteins involved in the yeast two-hybrid (Y2H) interaction. Red line refers to a very high-confidence Y2H interaction. All Y2H interactors are described in Supplementary file 2. Interactors are grouped according to the complex to which they belong.

**Appendix 1—figure 2.**
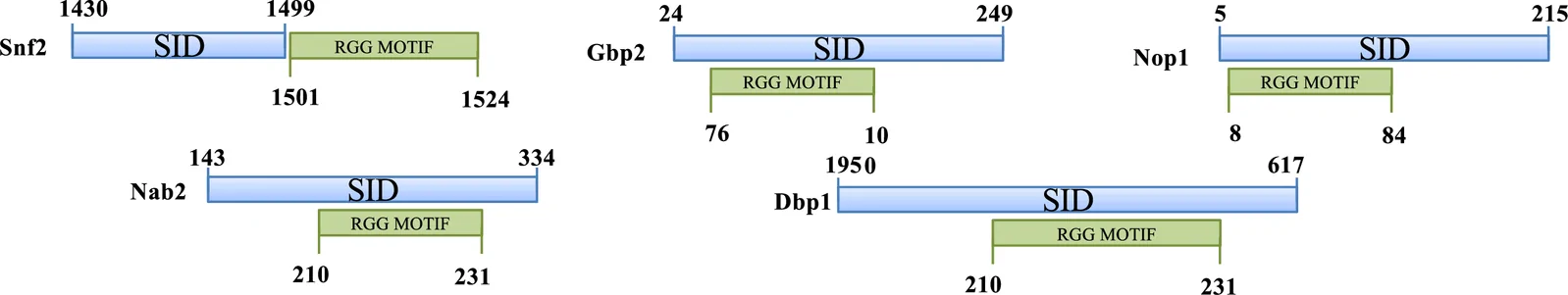
SID (blue) and RGG motif (green) within the RGG proteins. The interaction domains indicated represent the minimal overlapping DNA sequence present in multiple independent yeast two-hybrid (Y2H) interacting clones of the same gene. Each genomic fragment of a Y2H clone was analyzed, and the shared overlapping region for a given gene was determined to be the only common element among all interacting clones. As such, this region represents the minimal sequence required for interaction.

**Appendix 1—figure 3.**
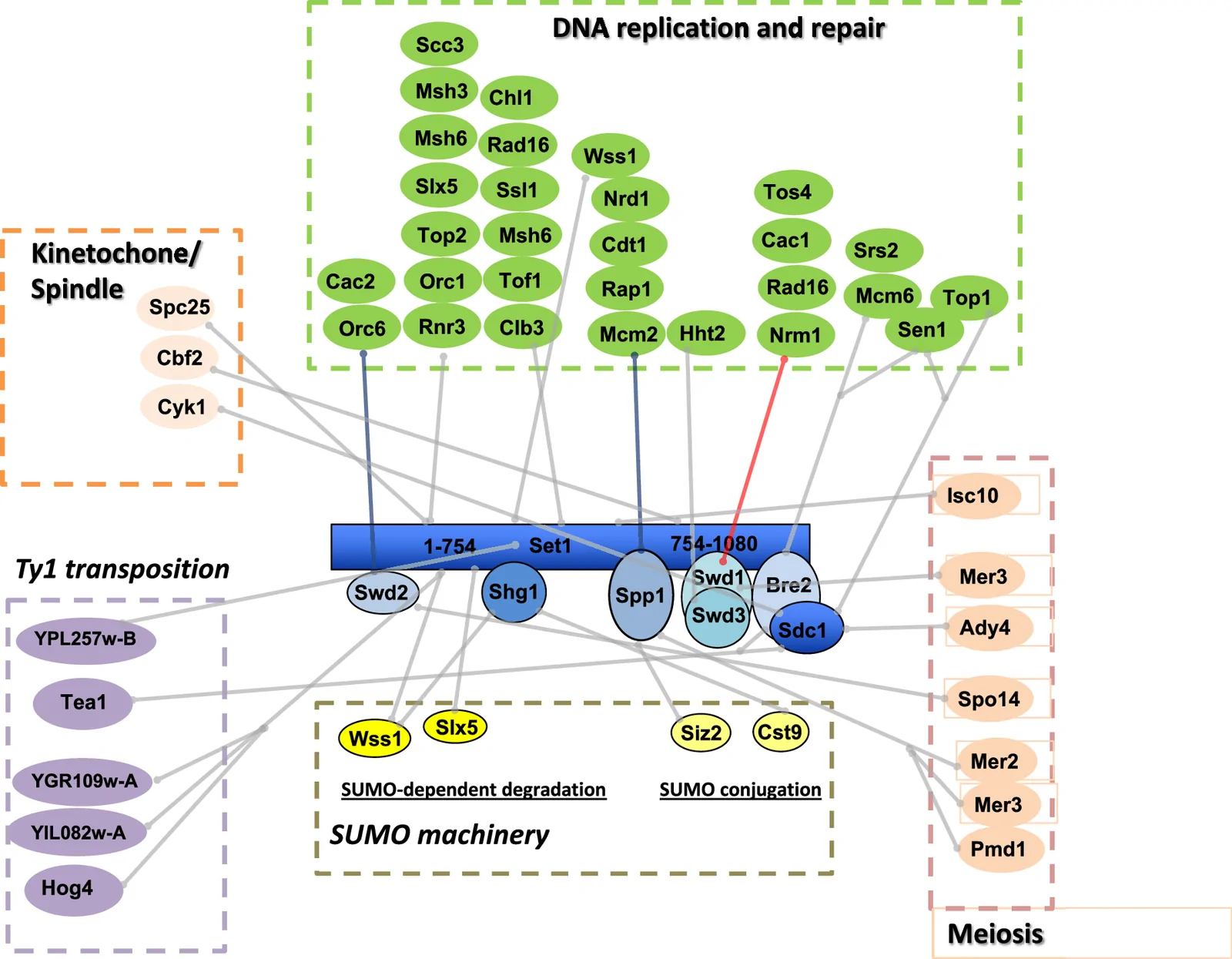
SET1C yeast two-hybrid (Y2H) interactors involved in DNA transactions. The processes in which Y2H interactors are involved are shown in the figure. Red and blue lines refer to the confidence of the Y2H interaction.

**Appendix 1—figure 4.**
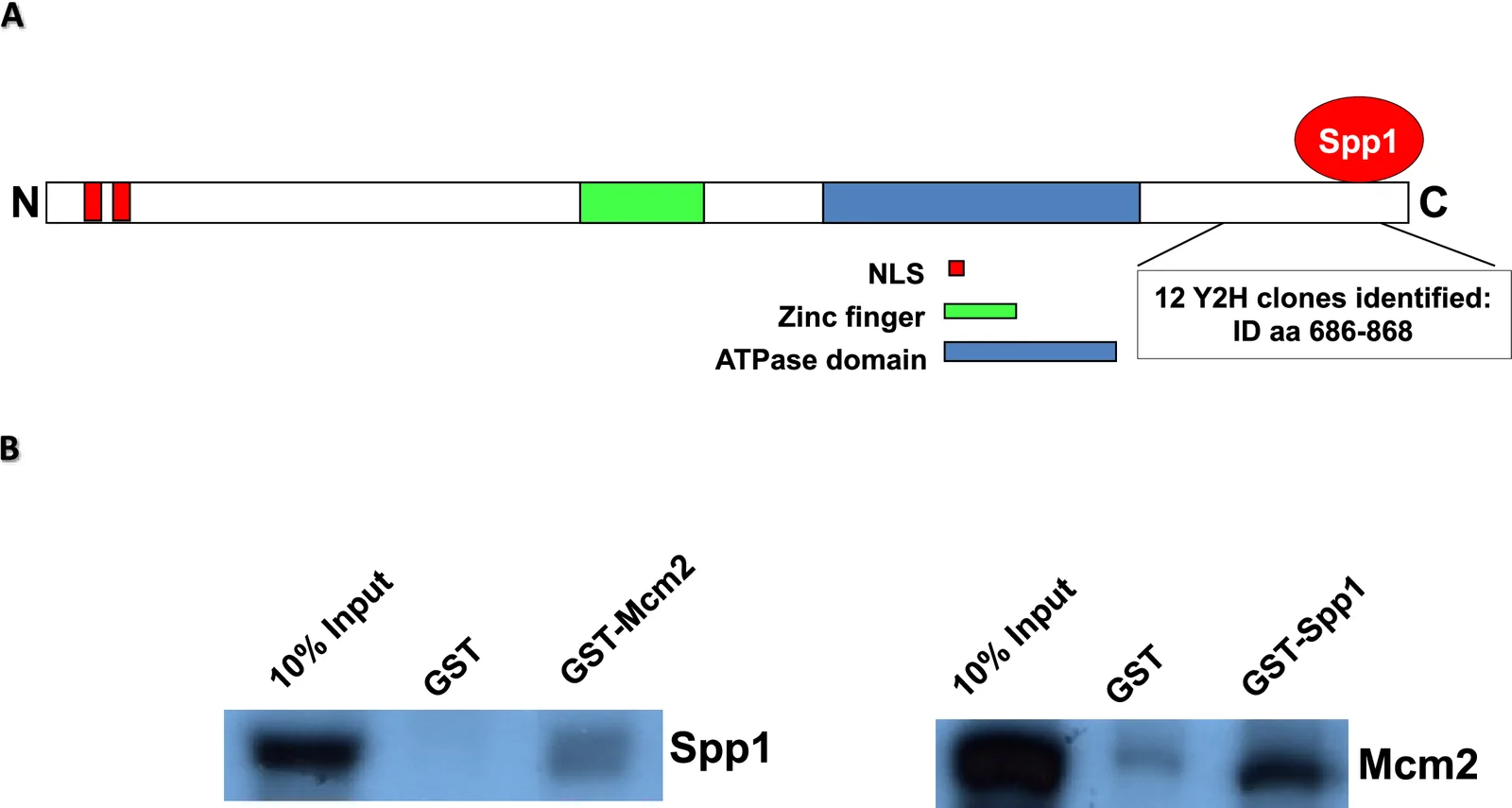
Spp1 interacts weakly with Mcm2 in vitro. (**A**) Schematic representation of Mcm2. The minimal region of interaction with Spp1 is located at the extreme C-terminus of Mcm2. (**B**) Pull-down assays were performed with GST, GST-Spp1, and GST-Mcm2 fusion proteins. ^35^S-labeled in vitro-translated proteins served as preys; input shows 10% of radioactive material included in binding reactions. Appendix 1—figure 4—source data 1.Source data for the autoradiography shown in Appendix 1—figure 4.

**Appendix 1—figure 5.**
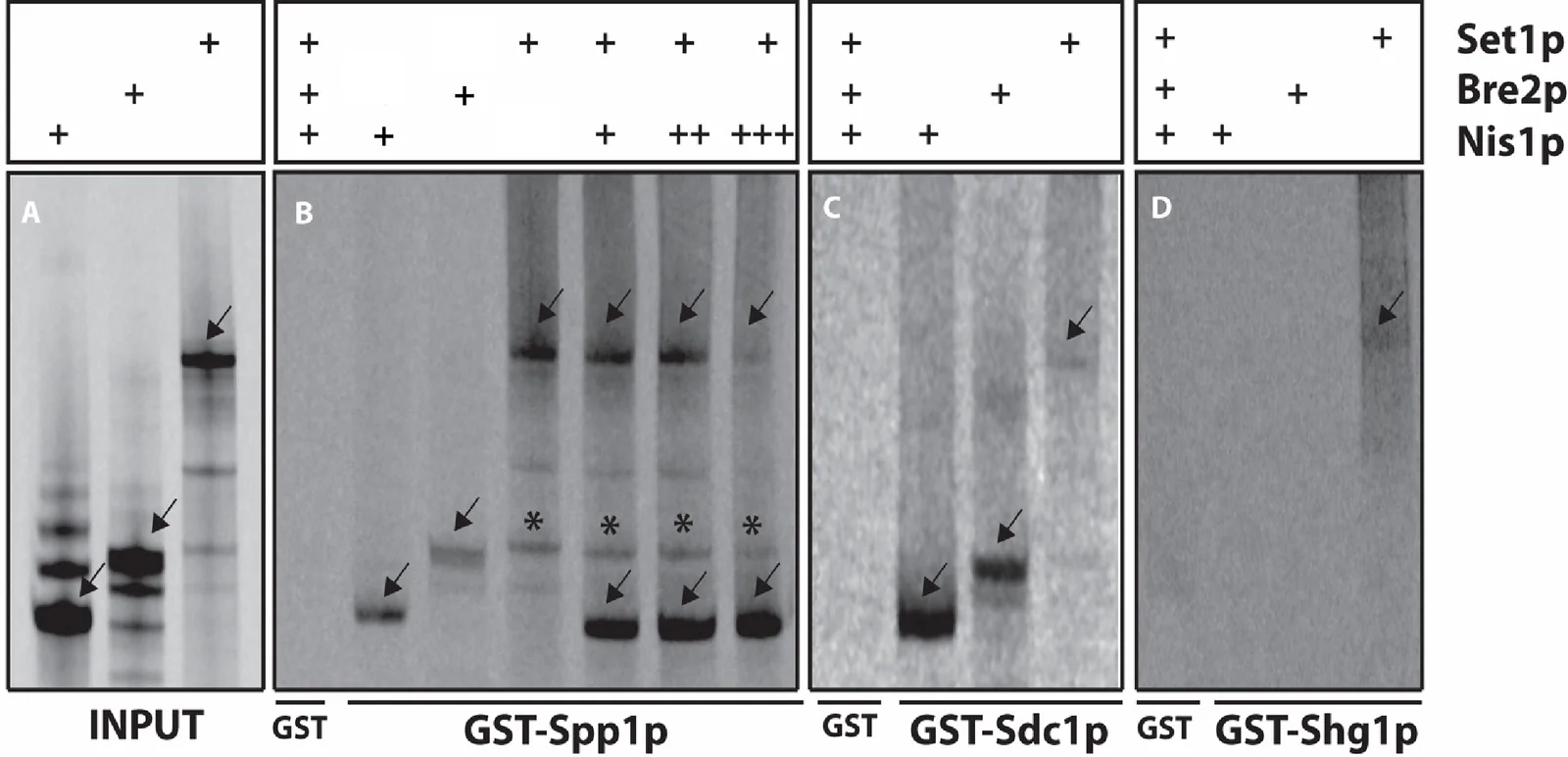
Spp1 and Sdc1 interact with Nis1 in vitro. Pull-down assays were performed with GST, GST-Spp1, GST-Sdc1, and GST-Shg1 fusion proteins as indicated at the bottom of the panels. ^35^S-labeled in vitro-translated proteins served as preys; input shows 10% of radioactive material included in binding reactions. The asterisk marks a band that is an undefined Set1 in vitro translation product, and it is marked so that it is not confused with full-length Bre2. The arrows point to the expected full-length in vitro translation products. Nis1 (46 KDa), Bre2 (58 KDa), Set1 (124 KDa). Appendix 1—figure 5—source data 1.Source data for the autoradiograph corresponding to Appendix 1—figure 5.

**Appendix 1—figure 6.**
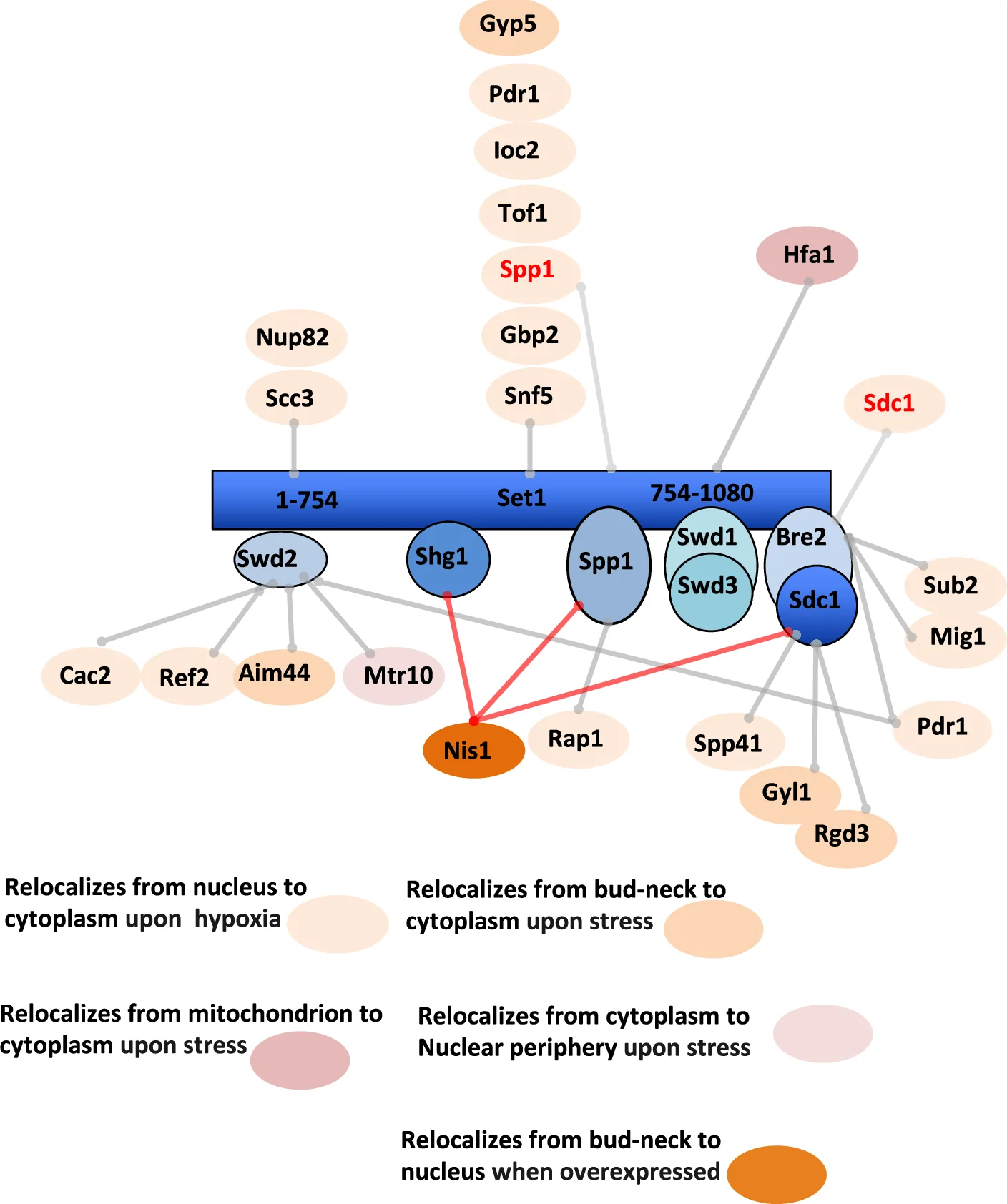
Identification of SET1C yeast two-hybrid (Y2H) interactors showing changes in localization following hypoxia. SET1C interactors that change localization are shown. In hypoxia, 243 proteins change their localization (Henke et al., 2011). Several components of the SWI/SNF complex were found to rapidly change location (from nucleus to cytosol) in hypoxia (Dastidar et al., 2012).

**Appendix 1—figure 7.**
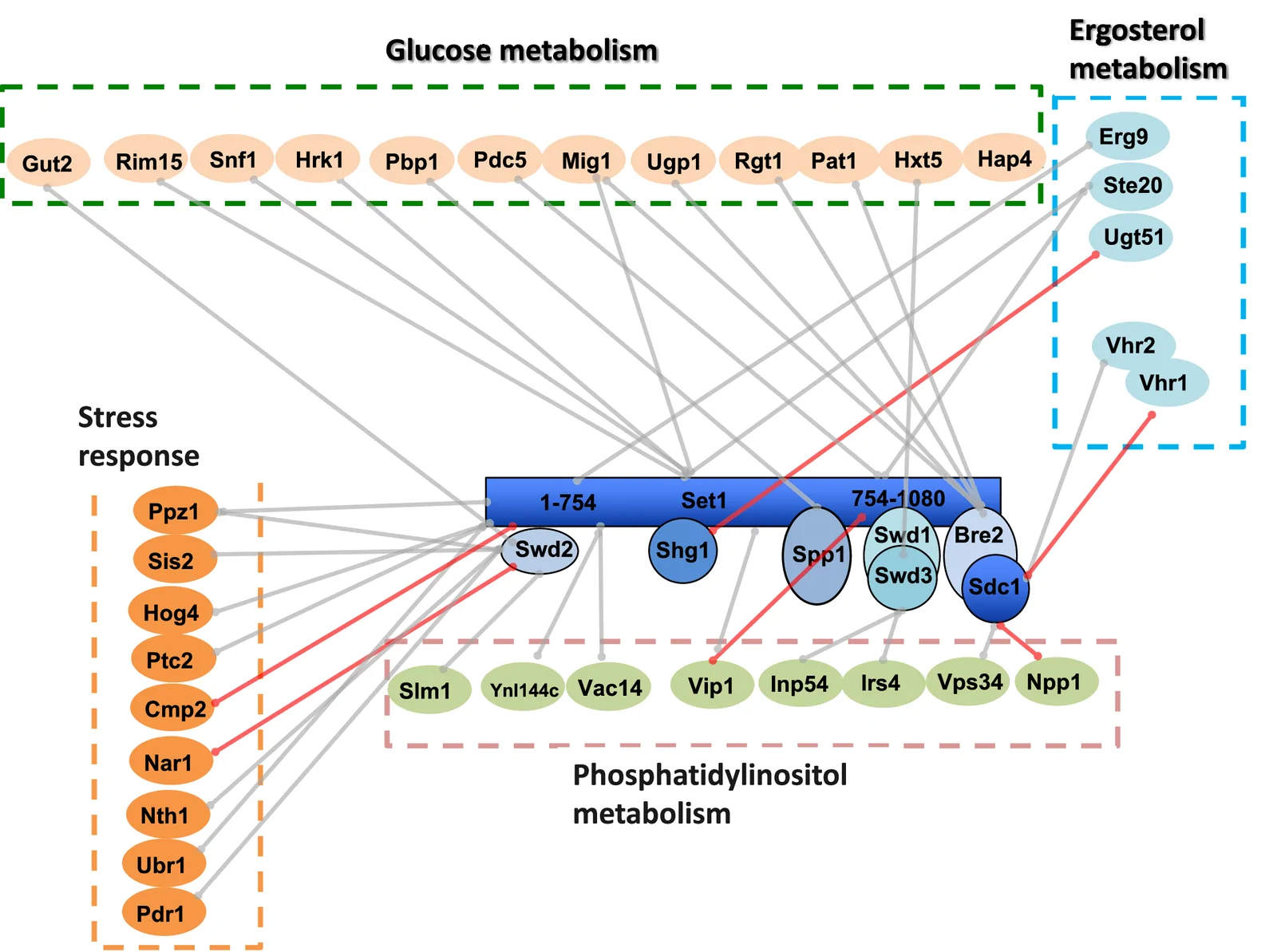
SET1C yeast two-hybrid (Y2H) interactors are involved in stress responses and metabolism pathways. The processes in which Y2H interactors are involved are shown in the figure. Red arrows indicate high-confidence Y2H interactions.

## Data Availability

Reagents are available upon request to the corresponding authors. The mass spectrometry proteomics data have been deposited to the ProteomeXchange Consortium (https://www.proteomexchange.org/) via the PRIDE partner repository (https://www.ebi.ac.uk/pride/): Snf2-B3 data (accession number PXD061448), Snf2-GFP complex (accession number PXD061496), Snf2 PTM (accession number PXD061531). Following the departure of the author responsible for Figure 9 from the laboratory to pursue other professional responsibilities, and the laboratory move to the IRCAN (Nice), the raw data associated with this figure could no longer be located. The following datasets were generated: AudebertS 2025Protein composition of the Snf2-GFP complexProteomeXchangePXD061496 KimYH ProteomeXchange2025Mass-spectrometry experiment design to identify the methylation sites of Snf2-B3PXD061448 AudebertS ProteomeXchange2025PTM in Snf2-GFP in WT and set1∆ strainsPXD061531

## Additional files

Supplementary file 1. Plasmids and yeast strains used in this study.

Supplementary file 2. All SET1C interactors identified in the 10 yeast two-hybrid (Y2H) screens. The interactors common to several subunits and to common Set1 fragments are shown on sheet 13 of the table.

Supplementary file 3. Mass spectrometry analysis of TAP-Nis1 affinity purification.

Supplementary file 4. H3K4-like domain proteins. We used the scansite search algorithm (http://scansite.mit.edu) and systematically identified sequence motifs that are related to the Set1C modification site in histone H3.

Supplementary file 5. Protein composition of the Snf2-GFP complex in WTand set1∆ strain.

Supplementary file 6. Snf2 post-translational modifications in either wild-type (WT) or *set1∆* strains.

MDAR checklist

## Appendix 1

**Appendix 1—table 1.** H3K4-­ like proteins. Shown are selected proteins containing sequences similar to the modification site found in histone H3. An exhaustive list can be found in Supplementary file 3.

protein	size (kDa)	motif	function
Histone H3	35	**ART** K **QT**	core histone protein required for chromatin assembly
Nrm1	57	**KT** K **QT**	transcriptional co-repressor of MBF-regulated gene expression
Nrd1	84	**RS** K **Q**	transcription termination and 3' end maturation of nonpolyadenylated RNAs
Rix1	107	**KT** K **QS**	processing of ITS2 sequences from 35 S pre-rRNA
Dbf2	86	**RT** K **Q**	transcription and stress response, part of a network of genes in exit from mitosis
Dbf20	86	**RT** K **Q**	late nuclear division, one of the mitotic exit network (MEN) proteins
Not5	86	**KT** K **Q**	transcription initiation, elongation and in mRNA degradation
Mcm2	120	**ART** K	DNA replication
Prp8	300	**RT** K **Q**	second catalytic step of splicing; mutations of human Prp8 cause retinitis pigmentosa
