# Supplementary material for: A systematic interactome of Saccharomyces cerevisiae SET1C expands its functional landscape and identifies candidate regulatory connections
Source: eLife. 2026 Sep 16;15:RP109886. doi: 10.7554/eLife.109886 (PMC13581290; doi:10.7554/eLife.109886)
Supplement: Peer review history

# Editor report: eLife Assessment

**DOI:** 10.7554/eLife.109886.3.sa0

**Authors:** Andrés Aguilera

**Affiliations:** CABIMER, Universidad de Sevilla Spain

**Keywords:** Incomplete, Valuable

This study uses the yeast two-hybrid assay to identify proteins that may interact with yeast Set1 and other subunits of COMPASS/Set1C, the histone H3K4 methyltransferase, providing also some evidence for Set1 sumoylation and a role of SET1C methylating other factors in vitro. The results are **valuable** and they should contribute to understanding the functions of the conserved SET1C complex, as they suggest potential functional connections with RNA biogenesis, chromatin remodeling, and non-histone methylation whose implications would yet need to be explored. Nevertheless, apart from the fact that only a small subset of the Y2H interactions is further examined, the validating experiments are only partial or inconclusive, the strength of evidence being **incomplete** at this point, although with improvements over the previous version.

# Referee report: Reviewer #1 (Public review)

**DOI:** 10.7554/eLife.109886.3.sa1

The manuscript has been improved in response to the reviewing. Although overinterpretation has been partially reduced compared to the previous version, the main concerns on the manuscript remain. The experiments have been conducted according to rigorous standards and the limitations of the results have been discussed to provide a comprehensive interpretation. However, this still represents an incomplete study in which the conclusions are insufficiently supported by the data provided.

# Referee report: Reviewer #2 (Public review)

**DOI:** 10.7554/eLife.109886.3.sa2

Summary:

This paper starts with a large-scale yeast two-hybrid (Y2H) screen using Set1 (full-length and smaller parts) and other Set1C/COMPASS subunits as bait. There are hundreds possible interactions identified, but only a small number are given any follow-up. While it's useful to document all the possible interactions, the unfocused and preliminary nature of the results makes the paper feel scattered and incomplete.

Strengths:

The Y2H screen was very comprehensive, producing lots of interesting possible leads for further experiments.

Weaknesses:

Most interactions were not further tested, and even in the case of those that were, the experiments are often inconclusive or incomplete.

# Referee report: Reviewer #3 (Public review)

**DOI:** 10.7554/eLife.109886.3.sa3

The SET1C/COMPASS complex is the histone H3K4 methyltransferase in *Saccharomyces cerevisiae*, where it plays pivotal roles in transcriptional regulation, DNA repair, and chromatin dynamics. While its canonical function in histone methylation is well-established, its full interactome remains poorly defined. Moreover, whether SET1C methylates non-histone substrates has been an open question.

In this study, Luciano et al. employ systematic yeast two-hybrid (Y2H) screening to uncover novel interactors and functions of SET1C. Their findings reveal potential functional connections to RNA biogenesis, chromatin remodeling, and non-histone methylation.

The authors performed multiple Y2H screens using Set1 (full-length, N-terminal, and C-terminal fragments) and each of its seven subunits as baits. They identified high-confidence interactors that link SET1C to diverse cellular processes, including chromatin regulation (e.g., the SWI/SNF complex via Snf2), DNA replication (e.g., Mcm2, Orc6), RNA biogenesis (e.g., spliceosome components Prp8 and Prp22; polyadenylation factors Pta1 and Ref2), tRNA processing (e.g., Trm1, Trm732), and nuclear import/export (e.g., importins Kap104 and Kap123). Some of these interactions were further validated by immunoprecipitation or in vitro assays.

Given the interaction of Set1 with Slx5 and Wss1-proteins involved in SUMO-dependent processes-the authors investigated and convincingly demonstrated that Set1 is sumoylated. This modification may influence the function and regulation of the SET1C complex.

Finally, the authors provide evidence that SET1C methylates Snf2, the catalytic subunit of the SWI/SNF chromatin remodeling complex.

One of the interactors, Nrm1, contains a domain resembling the H3K4-methylated sequence, which is also present in other proteins. Whether this H3K4-like domain is required for methylation remains to be demonstrated

Strengths:

This study offers valuable insights into the interactome of SET1C, suggesting potential links between the complex and a wide range of cellular processes. It also provides information on the possible regulation of Set1 by sumoylation. Finally, the finding that Snf2 is methylated in a Set1-dependent manner could significantly expand the known targets and functions of SET1C.

Weaknesses:

Many of the Y2H interactions remain to be validated and have to be considered as a starting point for further studies. Their functional significance remains to be explored. Several conclusions based on these 2HY data are speculative.

# Author comment: Author response

**DOI:** 10.7554/eLife.109886.3.sa4

**Authors:** Pierre Luciano, Kihyun Park, Stephane Audebert, Luc Camoin, Carlos A Niño, Da Kyeong Park, Isabella E Maudlin, Marion Dubarry, Lara Lee, Marlene Oeffinger, Jean D Beggs, Young Hye Kim, Jaehoon Kim, Bernhard Dichtl, Vincent Geli

**Affiliations:** Centre National de la Recherche Scientifique MARSEILLE France; Korea Advanced Institute of Science and Technology DAEJON Republic of Korea; Aix Marseille Universite, CNRS, INSERM, Institut Paoli-Calmettes Marseille France; Inserm Marseille France; Centre National de la Recherche Scientifique MARSEILLE France; Korea Basic Science Institute Science Institute Cheongju Republic of Korea; school of biological sciences Edinburgh United Kingdom; Centre National de la Recherche Scientifique MARSEILLE France; Centre National de la Recherche Scientifique MARSEILLE France; Instiut de recherches cliniques de Montréal Montréal Canada; school of biological sciences Edinburgh United Kingdom; Korea Basic Science Institute Science Institute Cheongju Republic of Korea; Korea Advanced Institute of Science and Technology Daejeon Republic of Korea; Deakin University Geelong Australia; Centre National de la Recherche Scientifique Marseille France

The following is the authors’ response to the original reviews.

**eLife Assessment**

This study uses the yeast two-hybrid assay to identify proteins that may interact with yeast Set1 and other subunits of COMPASS/Set1C, the histone H3K4 methyltransferase, providing also some evidence for Set1 sumoylation and a role of SET1C methylating other factors in vitro. The results are valuable, and they should contribute to understanding the functions of the conserved SET1C complex, as they suggest potential functional connections with RNA biogenesis, chromatin remodeling, and non-histone methylation, whose implications would yet need to be explored. Nevertheless, apart from the fact that only a small subset of the Y2H interactions is further examined, the validating experiments are only partial or inconclusive, the strength of evidence being at this point incomplete.

We present a systematic SET1C interaction map that provides a structured resource for generating and testing new hypotheses on SET1C function. We emphasise that these interactions represent a hypothesis generating resource rather than a set of validated protein–protein interactions. To reflect this, the manuscript has been carefully revised to distinguish clearly between observation and interpretation, and to avoid overstatement of the data. Accordingly, we have revised the title and the abstract. Selected examples are explored further to illustrate how candidates from the dataset can be followed up, but the primary contribution of this work is to provide a structured framework and resource that can guide future mechanistic studies of SET1C function.

We thank the reviewers for their thoughtful comments. We have followed their recommendations by modifying the structure of the manuscript, removing distracting results and relocating some figures to the supplementary materials to improve the readability of the manuscript. At the same time, the reviewers acknowledge that the dataset is extensive and that aspects of the validation work are valuable.

The changes made to the manuscript's structure in accordance with the reviewers' recommendations are as follows:

(1) Figure 1 is accompanied by a table (Table S2) with the raw data describing all the interactions from the ten 2H screens. This table also lists common interactors found in the independent screens. I'm afraid Table S2 was omitted from the initial submission of the manuscript

(2) Figure 2 has been modified to include an AlphaFold modeling of a seven-subunit Set1C complex (Set1– Bre2–Sdc1_2_–Swd1–Swd3–Spp1) together with Kap104. Figure 2D has been moved to a new Figure S2

(3) The initial figure S2, which was problematic, has been removed, along with the accompanying text.

(4) Figure 3 of the original paper has been moved to the supplementary material and is now shown as a new Figure S3.

(5) Figure 5 in the original paper becomes Figure 3 in the revised version

(6) Figure S3 (Co-IP between Set1 and Prp22), which serves as validation data, has been moved to the main figures and is now presented as Figure 4.

(7) Figure 6 in the original paper becomes Figure 5 in the revised version

(8) Figure 4 from the original paper has been repositioned as the first figure (new Figure 6) of the biochemical characterization of the interaction between Snf2 and Set1C.

(9) Figure 7 has been removed from the manuscript. We have kept the original Figure 7E as a new Figure S6.

(10) Figures 8, 9, 10 become Figures 7, 8, 9.

**Public Reviews:**

**Reviewer #1 (Public review):**

We thank Reviewer 1 for the careful and thoughtful evaluation of our manuscript. We fully agree that yeast two hybrid screening provides candidate interactions that require cautious interpretation, and we recognise that our original version did not always make this sufficiently explicit.

In the revised manuscript, we have made substantial changes to address this central concern. All Y2H interactions are now consistently presented as candidate or potential interactions, and speculative statements have been either removed or explicitly framed as hypotheses. Our intention is that the reader can clearly separate the dataset itself from any proposed biological implications.

Second, we have refocused the manuscript to better reflect its primary contribution. We now present the Y2H screens as a comprehensive resource that defines a set of candidate interactions for SET1C, rather than as a set of validated functional relationships. In line with this, we have reduced the emphasis on speculative models and removed sections where the connection to experimental evidence was not sufficiently strong. This includes the removal of Fig. S2 and Fig. 7 and the associated text, as well as the relocation of several figures to the supplementary material. Where appropriate, we have added statements highlighting the limitations of the approaches used and the need for future work to establish physiological relevance.

More generally, we agree with the reviewer that the value of Y2H data lies in generating testable hypotheses rather than establishing conclusions. We have therefore revised the manuscript throughout to ensure that the interpretation remains proportionate to the strength of the evidence.

We hope that these changes address the reviewer’s concerns and result in a clearer and more appropriately balanced presentation of the data.

The manuscript by Luciano et al is a collection of experiments about the yeast histone 3 lysine 4 methyltransferase, Set1, starting with 10 yeast two-hybrid screens (Y2H). Y2H screens were briefly popular 20+ years ago, but the persistently unfavourable false-to-true positive ratios limited their utility, and the conclusion emerged that Y2H is an unreliable approach for gathering protein-protein interaction data. Y2H outcomes are candidate interaction lists at best, strongly contaminated by false positives. Here, the authors employed a company (Hybridomics) to perform the Y2H screens.

The primary data is not presented, and the outcomes are summarized using the Hybridomics in-house quality scoring system in Figure 1A. It is not possible to evaluate these data, and the manuscript presents cartoon summaries that the reader must accept as valuable.

Hybrigenics brings extensive experience from conducting numerous screens, enabling the team to recognize recurring false positives that commonly arise in screening assays. In their detailed analysis, Hybrigenics reports the number of clones recovered and the extent of overlap among interaction regions, both of which contribute to the confidence scores they assign. Table S2, provided in the revised version, more accurately reflects the raw data obtained by Hybrigenics. Nevertheless, we agree that false positives contaminate the list of potential interactors. Some interactions may also be indirect through a common interactor and do not reflect a physiological interaction.

(1) Based on the extensive knowledge about Set1C/COMPASS acquired from genetics and biochemistry by many labs (including the Geli lab), the results presented here from the 10 Y2H screens are notably patchy. Of the 7 subunits of this complex, only one (Spp1) was identified using Set1 as bait. Conversely, as baits, Swd2, Spp1, Shg1, captured Set1, and the Bre2-Sdc1 interaction was reciprocally identified. These interactions were scored at the highest confidence level, which lends some confidence to the screens. However, the missing interactions, even at the third confidence level, indicate that any Y2H conclusions using these data must be qualified with caution. The authors do not appear to be cautious in their lengthy evaluations of these candidate interactions, which are illustrated with cartoons in Figures 2 and 3, with some support from the literature but almost without additional evidence. Snf2 is a particularly interesting candidate, which the authors support with pull-down experiments after mixing the two proteins in vitro (Figure 4). After Y2H, this is the least convincing evidence for a protein-protein interaction, and no further, more reliable evidence is supplied.

We thank the reviewer for raising this important point regarding the strength of the evidence supporting the Set1– Snf2 interaction. We agree that the current data do not establish a definitive physiological interaction. In the discussion, we explicitly note the limitations of the current data.

For Figure 2, as recommended by referee 2, we performed AlphaFold modeling of a seven-subunit Set1C complex (Set1–Bre2–Sdc1_2_–Swd1–Swd3–Spp1) together with Kap104. Consistent with the Y2H data, the model recapitulates binding of the Kap104 SID to the PY-NLS region of Set1 (residues 40–90).

We have moved Figure 3 in the supplementary materials.

(2) Figure 5 continues the cartoon summary of extrapolations from the Y2H screens, again without supporting evidence, except that the authors state.

Figure 5 is now Figure 3. We have added the statement in the text: “It is not feasible to validate all of these interactions within the limits of this manuscript, and their validity should therefore be interpreted with caution. Nonetheless, these findings provide a useful basis for future research”.

"We have refined the interaction region between Set1, Prp8 and Prp22, showing that Prp8 and Prp22 interact strongly with Set1-F4 (n-SET). Prp22 interacts in addition with Set1-F1 (Figure S2)." However, Figure S2 does not show this evidence and is incoherent.

When we say that we have refined the interaction region between Set1, Prp8, and Prp22, we mean that we have restricted the interaction regions according to Y2H criteria. Indeed, we have not shown the spots illustrating the results. This statement has been deleted as well as Fig. S2

The figure legends for Figure S2B and C do not correspond to the figure.

(B) Expression of the F1-F5 fragments in yeast cells. Fusion proteins were detected with an anti-GAL4 monoclonal antibody. TOTO yeast cells (Hybrigenics) were transformed with the different pB66-Set1-F1 to F5 plasmids and subsequently with either P6, pP6-Snf2 762-968, pP6-Prp8 37-250, or pP6-Prp22 379-763 that were identified in the Y2H screens. Transformed cells were incubated 3 days at 30{degree sign}C on SD-LEU-TRP and then restreaked on SD-LEU-TRP-HIS with 3AT. Cell growth was monitored after 2 days at 30{degree sign}C.

(C) Solid and dotted arrows indicate that transformed TOTO cells transformed with pB66-Set1-F1 to F5 and the indicated prey (Snf2, Prp8, and Prp22) are growing in the presence of 20 mM and 5 mM of AT, respectively.

Figure S2D is two almost featureless dark grey panels accompanied by the figure legend (D) Control experiment showing that TOTO cells transformed with p6 and pB66-Set1-F4 are not gowing (sic) in the presence of 5 mM or 20 mM AT.

We agree that the legend for Figure S2 was unclear and does not accurately describe the panels shown in the figure. Fig; S2 has been deleted in the revised version. The results shown in the original Fig. S2 add limited information and may detract from the clarity of the main points.

In the revised version, we have moved the CoIP analysis demonstrating the interaction between Set1 and Prp22 (previously shown in Figure S3) into the main figures (now Figure 4) to further support and validate the two-hybrid screening results presented there.

Line 343. Interestingly, the two-hybrid screens reveal that Set1 1-754 interacted with Gag capsid-like proteins of Ty1 (Figure S5), raising the possibility that Set1 binding to Ty1 mRNA is linked to the interaction of Set1 1-754 with Gag.

This is another example of the primary mistake repeatedly made by the authors -Y2H interactions are candidate results and not conclusive evidence.

This statement is supported by our previous findings showing that Set1 binds Ty1 mRNA independently of its dRRM domain and represses Ty1 mobility at a post-transcriptional stage (Luciano et al., Cell Discovery, 2017; PMID: 29071121). One possible explanation for Set1 association with Ty1 mRNA is its interaction with the Gag capsidlike protein. In this context, the observed interaction between Set1(1–754) and Gag capsid-like proteins is consistent with this model.

To further illustrate this point, the authors highlight the candidate interaction between Nis1 and 3 Set1C subunits.

While we agree that the Nis1-Set1C interaction has not been demonstrated beyond doubt, we feel that our Y2H and in vitro binding experiments provide reasonable evidence that the interactions may be relevant. It is important to consider that any interaction assay can provide negative (and false positive) results, this includes Y2H, in vitro binding and mass-spec analysis of purified complexes from cells. We feel that it is not appropriate to only trust protein interactions that are strong and stable enough to be demonstrated via purified complexes. It is clear that some protein interactions do occur in transient and weak manner and therefore are not compatible with biochemical purification approach. This indeed is the strength of alternative methods like Y2H and in vitro binding assays, that interactions can be identified and tested even if the physiological context of the interaction may be more complex.

(3) After multiple speculations based on the Y2H candidates, the authors changed to focus on sumoylation of Set1, which has previously reported to be sumoylated. Evidence identifying two sumoylation sites in Set1, in the N-SET and SET domains, is valuable and adds important progress to the role of sumoylation in the regulation of H3K4 methyltransferase, relevant for all eukaryotes. This illuminating part of the manuscript is only tenuously connected to the preceding Y2H screens and concomitant speculations.

We thank Referee 1 for their comment. While it is true that there is only a modest connection between Set1 interactors involved in direct or indirect sumoylation and the characterization of Set1 SUMOylation sites, we believe that this does not constitute a weakness of the manuscript.

(4) The manuscript then describes a red herring exercise involving Set1 methylation of Nrm1. In an already speculative and difficult manuscript, it is exasperating to read a paragraph about a failed idea. Apart from panel E, Figure 7 is a distraction, and I believe it should not be shared.

(5) However, despite the failure with Nrm1, Line 443 - The H3K4-like domain in Nrm1 raised our attention to other yeast proteins that carry such sequences.

This line of thinking is even less connected to the Y2H screens than the sumoylation work.

However, the authors present a reasonable evaluation of the yeast proteome screened for six amino acids similar to the known H3K4 motif ARTKQT (Figure 7e).

(6) However, this evaluation goes nowhere and has no connection with the next section of the manuscript, which is entirely speculation about the regulation of metabolism and stress responses based on the Y2H results and selected evidence from the literature.

In response to comments 4 and 5, we have removed Fig. 7 and the paragraph titled “The transcriptional corepressor Nrm1 interacts with SET1C.” Part of this paragraph and the section describing the screen of the yeast proteome for six–amino acid sequences resembling the H3K4 motif (ARTKQT) has been kept as Fig. S6.

In the abstract, we have removed the sentence: We demonstrate that the transcriptional corepressor Nrm1 is methylated by SET1C in vitro suggesting that H3K4-like domains may represent a class of non-histone substrates for SET1C.

At the end of the introduction, we have deleted “the transcriptional corepressor Nrm1” in the sentence: In addition, we demonstrate that the transcriptional corepressor Nrm1 and the Snf2 AT-hook are both methylated by SET1C in vitro

(7) The manuscript then describes more failed experiments regarding lysine methylation of Snf2 by Set1C, which unexpectedly reports arginine methylation rather than lysine. The manuscript does not currently meet the standard expected for this type of paper - the composition is somewhat incoherent and there are no previous reports of arginine methylation by SET domain proteins.

We have integrated extensive in vitro reconstruction experiments with complementary in vivo studies, all conducted according to the rigorous standards expected by leading journals. These approaches have allowed us to reach the conclusions presented in this manuscript. While some of these findings are unexpected, they are supported by the data. We have carefully discussed the results and their limitations to provide a comprehensive interpretation.

The manuscript presents a very experienced grasp of the literature and a sophisticated appreciation of the forefront issues, but a surprising failure to eliminate uninformative failures and peripheral distractions. The over interpretation of Y2H results is a dominating failure. There are some valuable parts within this manuscript, and hopefully, the authors can reformat to eliminate the defects and appropriately qualify the candidate data.

We thank Referee 1 for these insightful comments. In the revised version, we have followed the advice to remove non-informative failures and peripheral distractions. Additionally, we exercise greater caution to avoid over-interpreting the Y2H results.

**Reviewer #2 (Public review):**

Summary:

This paper starts with a large-scale yeast two-hybrid (Y2H) screen using Set1 (full-length and smaller parts) and other Set1C/COMPASS subunits as bait. There are hundreds of possible interactions identified, but only a small number are given any follow-up. While it's useful to document all the possible interactions, the unfocused and preliminary nature of the results makes the paper feel scattered and incomplete.

Strengths:

The Y2H screen was very comprehensive, producing lots of interesting possible leads for further experiments.

Weaknesses:

The results are useful but incomplete because only a small subset of the Y2H interactions is further examined. Even in the case of those that were further tested, the validating experiments are only partial or inconclusive.

Referee 2’s comments align in some respects with those of Referee 1. In the revised version, we have followed the detailed Referee 2 suggestions to reduce the scattered nature of the manuscript. In addition, we include an AlphaFold model of the interaction between the Set1 N-term 1-754 with the SID domain of Kap104 that involves the proposed Set1 PY-NLS sequence.

**Reviewer #3 (Public review):**

The SET1C/COMPASS complex is the histone H3K4 methyltransferase in *Saccharomyces cerevisiae*, where it plays pivotal roles in transcriptional regulation, DNA repair, and chromatin dynamics. While its canonical function in histone methylation is well-established, its full interactome remains poorly defined. Moreover, whether SET1C methylates non-histone substrates has been an open question. In this study, Luciano et al. employ systematic yeast two-hybrid (Y2H) screening to uncover novel interactors and functions of SET1C. Their findings reveal potential functional connections to RNA biogenesis, chromatin remodeling, and non-histone methylation.

The authors performed multiple Y2H screens using Set1 (full-length, N-terminal, and C-terminal fragments) and each of its seven subunits as baits. They identified high-confidence interactors that link SET1C to diverse cellular processes, including chromatin regulation (e.g., the SWI/SNF complex via Snf2), DNA replication (e.g., Mcm2, Orc6), RNA biogenesis (e.g., spliceosome components Prp8 and Prp22; polyadenylation factors Pta1 and Ref2), tRNA processing (e.g., Trm1, Trm732), and nuclear import/export (e.g., importins Kap104 and Kap123). Some of these interactions were further validated by immunoprecipitation or in vitro assays.

Given the interaction of Set1 with Slx5 and Wss1 - proteins involved in SUMO-dependent processes - the authors investigated and convincingly demonstrated that Set1 is sumoylated. This modification may influence the function and regulation of the SET1C complex.

Finally, the authors provide evidence that SET1C methylates proteins beyond histone H3K4, notably Nrm1, a transcriptional corepressor, and Snf2, the catalytic subunit of the SWI/SNF chromatin remodeling complex. Although Nrm1 contains a domain resembling the H3K4-methylated sequence (H3K4-like domain), this region does not appear to be required for its methylation. The search for other proteins containing similar domains as potential methylation candidates (p.12, first paragraph) seems less justified, given the lack of evidence supporting the requirement for the H3K4-like domain in methylation.

This study offers valuable insights into the interactome of SET1C, suggesting potential links between the complex and a wide range of cellular processes. However, the functional implications of the Y2H interactions remain to be explored further. Additionally, the study provides intriguing information on the possible regulation of Set1 by sumoylation. The discovery of Nrm1 and Snf2 as methylation substrates could significantly expand the known targets and functions of SET1C.

The results are supported by high-quality data.

We thank referee 3 for their positive comments

**Recommendations for the authors:**

**Reviewer #1 (Recommendations for the authors):**

Restructure the manuscript into at least two papers.

We thank the reviewer for this suggestion. In the revised manuscript, we have addressed this concern by substantially restructuring and streamlining the presentation. We consider the dataset, validation experiments, and functional observations to be closely integrated, and we believe that presenting them together provides the most coherent and impactful account of the work.

Minor points

There are several basic flaws in the manuscript that I feel indicate the co-authors have not proofread the manuscript sufficiently - 4 examples from early in the manuscript are listed below.

(1) The reference for Hybridomics is (73) - obviously from an earlier version that used a different referencing system that has not been corrected.

Thank you. This has been corrected.

(2) Line 194 - 197. These screens have proven their power and effectiveness. In particular, they identified ...... the CTD of Rpb1 as an interactor of the N-terminal region of Set1 (Bae et al, 2020) (Figure S1). Rbp1 interaction is not identified in the screens presented here, and Figure S1 is a cartoon and not primary evidence.

The interaction between the CTD of Rpb1 (Rpo21) and Set1 is reported in Table S2. The detailed characterization presented in Bae et al. (2020) was subsequently carried out as a direct follow-up to this screen.

(3) Line 205-211. The highly confident interactors of the seven SET1C subunits are shown in Figure 1C-E. We found that Spp1, Shg1 and Swd2 interact alone with Set1 (Figure 1C). The minimum Set1 region for which an interaction is found for each of these 3 subunits is shown in Figure 1C. The high confidence interactors of the seven SET1C subunits are shown in Figure 1C-E. We found that Spp1, Shg1 and Swd2 display Y2H interactions with Set1 (Figure 1C). The high confidence interactors of Spp1, Shg1 and Swd2 are indicated in Figure 1D (see also Table S2).

It is possible that Table S2 was omitted from the original submission, as it was requested during the production stage.

(4) Line 335. We have classified all Set1 and subunit interactors according to these SET1C roles (Figure S5). However, this refers to Figure S4 - many further references to Figure S5 are also to Figure S4.

Thank you. This has been corrected.

**Reviewer #2 (Recommendations for the authors):**

General recommendations:

(1) Figures 1, 2, 3, and 5 and their associated main text are essentially just lists of interactors, put in graphic form and grouped to allow speculation about possible biological functions for the interactions. But almost none of the ideas are tested, so these sections take much more space than warranted. Having so much preliminary Y2H data actually distracts attention from the follow-up experiments that are shown. I would move most or all of this to the supplement, consolidating the Y2H results into fewer figures (or even just the Table).

As mentioned earlier, the manuscript has been reorganized and Table S2 is provided.

(2) The Snf2 interaction gets the most follow-up, so separating Figure 4 from Figures 8-10 broke the flow of that story. I would group these figures together since all are related to the Snf2 AT hook story.

This was done accordingly.

(3) I understand that it's impossible to validate all the possible interactions, particularly if resources are limited. However, at least for the interactions that get further attention, it could be very useful to try some AlphaFold multimer predictions. A high confidence AlphaFold score would provide a second orthogonal piece of evidence to support the Y2H results.

We generated an AlphaFold model (Figure 2C) that recapitulates the key predictions for the Set1-Kap104 Y2H interaction.

Comments on specific sections:

(1) Y2H results. The text says Figure 1 shows all the high-confidence interactors. But the Set1 NTD interaction with the Rpb1 CTD is not shown here (it's in the supplement).

In Table S2, an interaction is observed between full-length Set1 and the Rpb1-CTD (14 repeats), where Rpb1 is referred to as Rpo21.

Figure 2 shows additional high-confidence interactors that do not appear in Figure 1, while others (like the Shg1Mog1 interaction) are shown in both Figures 1 and 2. It's confusing to scatter the data like this, which is why I recommend consolidating into a single figure or table.

In Figure 2, the high-confidence interactors of Set1 (1–754) are highlighted in red and green (Snf2, Gbp2, and Kap104), and all are also present in Figure 1. Dbp1, identified as a high-confidence interactor of Spp1, likewise appears in Figure 1. Table S2 summarizes all of these interactions.

(2) Line 219. How does a "high confidence" Set1-Kap104 Y2H interaction suggest the interaction is direct? Couldn't an indirect interaction also be tight and reproducible? This is an example where it would be worth seeing if AlphaFold also predicts an interaction and, if so, whether it involves the proposed NLS sequences.

Y2H screening indicated that Kap104 binds to the N-terminal region (aa 1–754) of Set1 via its Set1 interaction domain (SID). To validate this, we used AlphaFold to model the seven-subunit Set1C complex (Set1-Bre2-Sdc1(x2) Swd1-Swd3-Spp1) with Kap104. The resulting model showed borderline confidence for the overall fold (pTM = 0.53) and low confidence in subunit positioning (ipTM = 0.5). Visualization in PyMOL confirmed Kap104 SID binding to Set1(1–754), consistent with Y2H results. The structure highlights Kap104 SID interaction with Set1’s PY-NLS at residues 40–90; the second PY-NLS is neither visible nor engaged in this model.

(3) In the discussion of nuclear import interactors, what does it mean to say the Shg1-Mog1 interaction is "along the same line" as Set1-Kap104?

We meant that the interaction between Shg1 and Mog1 represents another example of an interaction between a Set1C subunit and a protein involved in nuclear import. Along the same line has been deleted in the revised version.

(4) To follow up on the Swd1-Nrm1 Y2H interaction, the paper shows that Nrm1 is methylated by Set1 in vitro (Figure 7), but it's not clear whether this has any biological significance. Without any in vivo follow-up, this figure is probably more appropriate for the Supplement.

As noted above, Figure 7 has been removed, only panel E of Figure 7 is retained in the revised version.

(5) Figures 6 and S8 show that Set1 is SUMOylated. Although it's not clear what this does to Set1 function or which E3 is responsible, the modification data looks convincing. The legend to Figures 6A and B says the Elutes samples are purified on nickel columns. Why are the Myc-Set1 and GB-Set1 proteins without the his-SUMO modification also binding to the nickel column? That's not happening in panels C and D. In the blots on the right for his-SUMO, is there any way to show that one of those bands is Set1? Maybe IP for MYC and then probe for the His tag?

We thank the reviewer for this observation. His-SUMO purification using Nickel beads was used to purify HisSUMOylated proteins. Purified proteins were analyzed by Western blot using anti-MYC or anti-GAL4 antibodies to detect SET1-His-SUMO, as well as anti-His antibodies to confirm the presence of purified His-SUMOylated proteins. As mentioned by the reviewer, we detected unmodified MYC-Set1 and GAL4-Set1 in both the (-) and (+) His-SUMO eluates. This phenomenon is most likely due to the stickiness of unmodified Set1 to the beads. This is a commonly observed phenomenon in this type of biochemical assay, particularly when analyzing large proteins such as Set1 (124 kDa). This stickiness behavior has been observed in similar SUMOylation assays, e.g., for Hpr1 (88 kDa) (Bretes H, 2014. PMID: 24500206), Nup1 (114 kDa), and Nup2 (78 kDa) (Folz H, 2019. PMID: 30837289). This stickiness was not observed when using Set1 fragments (panels C and D), most likely because the fragments lost the stickiness to the beads, a characteristic belonging only to the full-length Set1. We mention this point in the legend of the new figure 5.

(6) The Snf2 interaction gets the most follow-up. The GST pulldown validation of Set1 interaction with Snf2 AThook looks pretty good. However, the RGG repeats are necessary for the Set1 interaction with recombinant Snf2 proteins, but not for the co-IP of in vivo material. Again, AlphaFold could lend further support here.

Thank you for this helpful suggestion. We agree that structural modelling could, in principle, provide an additional and orthogonal line of support for the Set1-Snf2 interaction. We did explore this using AlphaFold. However, both Set1 and Snf2 contain extensive intrinsically disordered regions, including the regions implicated in the interaction, and none of the models we obtained provided interpretable structural insight into the interaction interface. In particular, the predicted complexes showed low confidence in relative domain positioning, which limits their usefulness for supporting or refining the interaction model. One possible explanation is that additional components are required to stabilise a meaningful interaction *in silico*. While we modelled Set1 within a seven-subunit Set1C complex, Snf2 was necessarily included in isolation from its native context. Given that Snf2 functions as part of multiple, heterogeneous chromatin remodelling complexes, the absence of its physiological binding partners may prevent AlphaFold from resolving a relevant interaction interface. In light of these limitations, we have not included the AlphaFold models in the manuscript, as we felt they would not provide reliable or informative support. Instead, we have focused on the experimental evidence presented. We have clarified this point in the revised discussion to acknowledge both the potential and the current limitations of structural prediction approaches in this context.

(7) The Snf2 methylation by Set1 is less convincing, and its biological significance is still unclear. I think it's pretty unlikely that Set1 could methylate arginine. The mass spectrometry is used for in vivo validation (mass spec), but mutating the lysines (Figure S11, S12) or Set1 deletion (Figure S14) doesn't seem to affect the signal. Could there be quantitative differences? Is there any way to quantitate the mass spec data to estimate the modified/unmodified ratio?

We thank the reviewer for highlighting the unexpected nature of the methylation results. We agree that the observation of arginine methylation in this context is surprising, particularly given that SET domain proteins are classically associated with lysine methylation. This is why we performed multiple in vitro and in vivo experiments, and careful interpretation data that were clear led us to conclude that Set1C methylates the arginines within the ARTSTRGR motif of the AT-hook. We agree that the biological significance of this modification remains unclear. We obtained data showing that deletion of the SID domain of Snf2 impairs yeast growth on lactate, whereas this mutant grows normally on glucose and galactose, in contrast to the Snf2Δ mutant, which exhibits poor growth on both glucose and galactose. In comparison, deletion of the RG motif of Snf2 does not affect growth on lactate. These results provide insight into the interaction between Set1 and Snf2 but do not shed light on the potential importance of methylation of the RG motif. We therefore chose not to include them. In the discussion, we acknowledge the limitations of the current evidence. Our intention is to retain these findings as potentially interesting observations while ensuring that their interpretation remains appropriately cautious.

Minor comments:

(1) Lines 153 and 163: Stress response is listed twice, but with different references. Maybe these need to be further defined or else combined?

We have deleted stress response line 163 and moved the references “Deshpande et al, 2022 and Nadal-Ribelles et al, 2015” line 153.

(2) Line 193: better to say the proteins were fused to the C- or N-terminus (rather than upstream/downstream). It would be worth mentioning if there was a reason why Swd2 was fused to the N-terminus, unlike all the others.

This has been done accordingly. In our hands, C-terminal fusions of Swd2 are not functional.

(3) Is the scoring scheme (highest, high, good) that produces the colors in Figure 1 shown in the table? It doesn't say what the tan color (two of the Bre2 interactors) means.

It is a mistake, Tea1 should be blue and Swi1 should not appear here. This has been fixed.

(4) Line 206. It's not clear what it means to say that three of the subunits "interact alone with Set1". It can't mean they only interact with Set1, since other interactors are shown in Figure 1B. If it meant to say the interactions don't require other COMPASS subunits? I don't see how you can tell that from the Y2H assay. Please clarify.

It means that these 3 subunits interact directly with Set1 without the need of another subunit, unlike of the other subunits.

(5) Line 252. While discussing the Set1 - Snf2 interaction, the paper cites Hirschhorn et al. That paper talks about Swi-Snf, but doesn't mention Set1 anywhere. Maybe the authors meant to cite a different paper?

We agree, this reference is not appropriated. It has been deleted.

(6) Figure S2 panels A and C are redundant and could easily be combined.

Figure S2 has been deleted.

(7) Figure S4: Should the green category also include transcription? Ssl1 is a TFIIH subunit, which could be involved in either transcription initiation or NER. Sen1 and Nrd1 are transcription termination factors, although Sen1 may also function in R-loop resolution.

We agree but it is already complicated as it is.
